# The medicinal thiosulfinates from garlic and *Petiveria* are not radical-trapping antioxidants in liposomes and cells, but lipophilic analogs are†
†Electronic supplementary information (ESI) available: additional figures referred to in the text, experimental procedures and full compound characterization, including NMR spectra. See DOI: 10.1039/c5sc02270c


**DOI:** 10.1039/c5sc02270c

**Published:** 2015-07-29

**Authors:** Bo Li, Feng Zheng, Jean-Philippe R. Chauvin, Derek A. Pratt

**Affiliations:** a Department of Chemistry and Biomolecular Sciences , University of Ottawa , 10 Marie Curie Pvt. , Ottawa , Ontario , Canada . Email: dpratt@uottawa.ca ; Fax: +1-613-562-5170 ; Tel: +1-613-562-5800 ext. 2119

## Abstract

The radical-trapping antioxidant (RTA) activities of allicin and petivericin, thiosulfinates widely believed responsible for the medicinal properties of garlic and *Petiveria*, were determined in phosphatidylcholine lipid bilayers. The results indicate that both compounds are surprisingly ineffective, in sharp contrast with previous studies in organic solution which showed that they undergo facile Cope elimination to produce sulfenic acids – potent radical-trapping agents. In an effort to understand the medium dependence of this activity, a more lipophilic (hexylated) analog of petivericin was synthesized and shown to be among the most effective RTAs known, but only in the presence of a hydrophilic thiol (*e.g. N*-acetylcysteine). Additional symmetric and unsymmetric thiosulfinates were synthesized to shed light on the structural features that underlie this reactivity. These studies reveal that amphiphilic thiosulfinates which undergo *S*-thiolation with a hydrophilic thiol to give lipophilic sulfenic acids are required, and that an activated methylene group – key to promote Cope elimination – is not. Interestingly, the added thiol was also found to regenerate the sulfenic acid following its reaction with peroxyl radicals. This activity was diminished at more acidic pH, suggesting that it occurs by electron transfer from the thiolate. Allicin, petivericin and hexylated petivericin were assayed as inhibitors of lipid peroxidation in human TF1a erythroblasts and HEK-293 kidney cells, revealing similar efficacies in the low μM range – the same range in which allicin and petivericin were found to induce cell death concomitant with, or as a result of, glutathione (GSH) depletion. In contrast, hexylated petivericin was not cytotoxic throughout the concentration range assayed, and had no effect on GSH levels. Taken together, the results in lipid bilayers and in cell culture suggest that the greater lipophilicity of hexylated petivericin enables it to partition to membranous cell compartments where it forms a lipid-soluble sulfenic acid that traps peroxyl radicals, whereas allicin and petivericin partition to the cytosol where they deplete GSH and induce cell death.

## Abstract 2

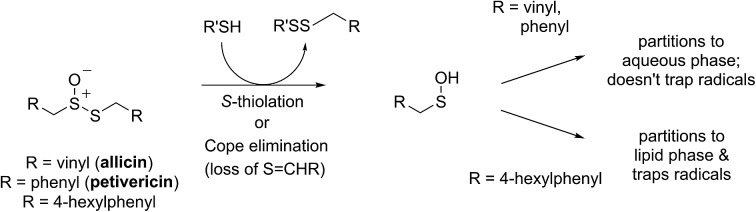
The radical-trapping antioxidant (RTA) activities of allicin and petivericin, thiosulfinates widely believed responsible for the medicinal properties of garlic and *Petiveria*, were determined in phosphatidylcholine lipid bilayers and mammalian cell culture.

## Introduction

The health benefits of garlic have been recognized since at least 2000 BC, making it the world's oldest known medicine.^1^ The medicinal properties of garlic, and other species of the allium genus, are widely attributed to their odorous organosulfur compounds. Of these, allicin (**1**) – the predominant thiosulfinate in garlic – is the most prominent example. Since its isolation by Cavallito and Bailey in 1944,^3^,^4^ allicin has demonstrated biocidal activities against several types of microorganisms, including bacteria, yeast and fungi.^5^,^6^ However, more recent interest has focused on allicin as a chemopreventive agent against cardiovascular^7^–^10^ and neurodegenerative^11^,^12^ disease as well as cancer.^13^–^16^ 
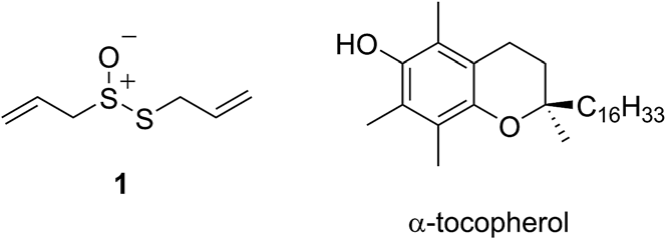


The medicinal properties of allicin are often ascribed to its controversial ‘antioxidant’ activity.^17^–^20^ The controversy surrounds the fact that while many reports show that allicin traps radicals or induces phase II antioxidant enzymes, a similar number of reports suggest that it is highly cytotoxic. In early work, allicin was shown to inhibit lipid peroxidation in liver homogenates by scavenging hydroxyl radicals in a concentration-dependent manner^21^ and a rate constant for its reaction with hydroxyl radicals was estimated to be 2 × 10^8^ M^–1^ s^–1^.^22^ However, as others have already correctly noted,^23^ essentially all organic molecules react with hydroxyl radicals at or near diffusion controlled rates, making it unlikely that this reactivity underlies allicin's biological activities. The trapping of peroxyl radicals is far more relevant as they react relatively discriminately,^24^,^25^ carrying the chain reaction that peroxidizes lipids to products that have been implicated in degenerative disease onset and progression.^26^–^28^ Okada *et al.* reported that allicin reacts with lipid-derived peroxyl radicals with a rate constant of 1.6 × 10^5^ M^–1^ s^–1^,^29^ suggesting it is a good radical-trapping antioxidant (RTA) – only 20-fold less reactive than α-tocopherol (α-TOH, 3.2 × 10^6^ M^–1^ s^–1^),^30^ the most potent form of vitamin E and Nature's premier lipophilic RTA.^31^

Intrigued by the high reactivity of allicin toward peroxyl radicals despite the fact that it is devoid of any of the structural features common to good RTAs (*e.g.* an electron-rich phenolic moiety such as in α-TOH),^31^,^32^ we investigated the mechanism of peroxyl radical-trapping by allicin. We demonstrated that the RTA activity was not due to allicin, but instead the 2-propenesulfenic acid formed *via* Cope elimination from allicin (eqn (1) and (2)).^33^ The same mechanism was demonstrated for petivericin (**2**), the analogous thiosulfinate derived from *Petiveria alliacea* – also known as the medicinal plant anamu found in South and Central America.^34^ While the 2-propenesulfenic acid derived from **1** and the phenylmethanesulfenic acid derived from **2** were too labile to study directly, investigations with the persistent 9-triptycenesulfenic acid **3** revealed that sulfenic acids have very weak O–H bonds (68–72 kcal mol^–1^),^35^ and undergo very fast reactions with peroxyl radicals (*k*_2_ = 3 × 10^7^ M^–1^ s^–1^).^36^
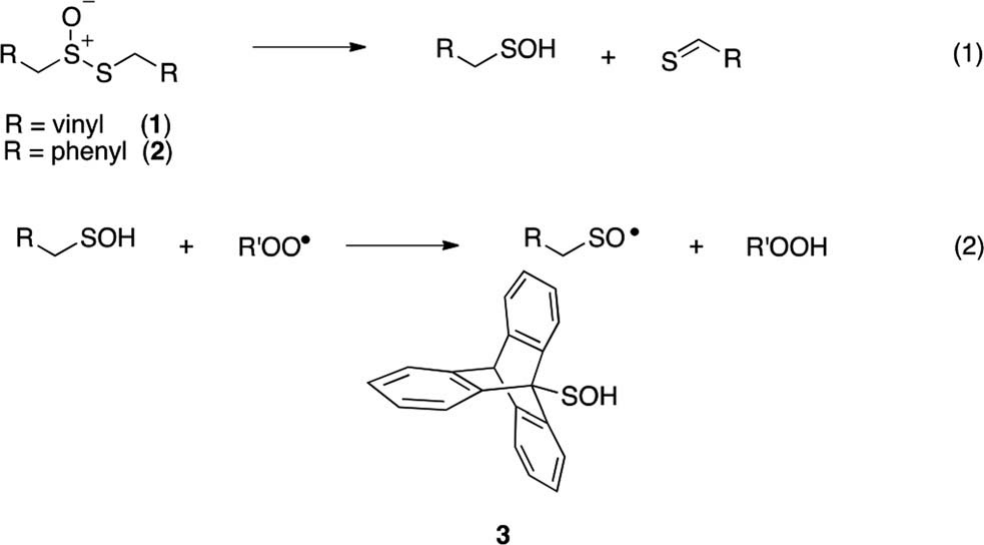


**Scheme 1:**
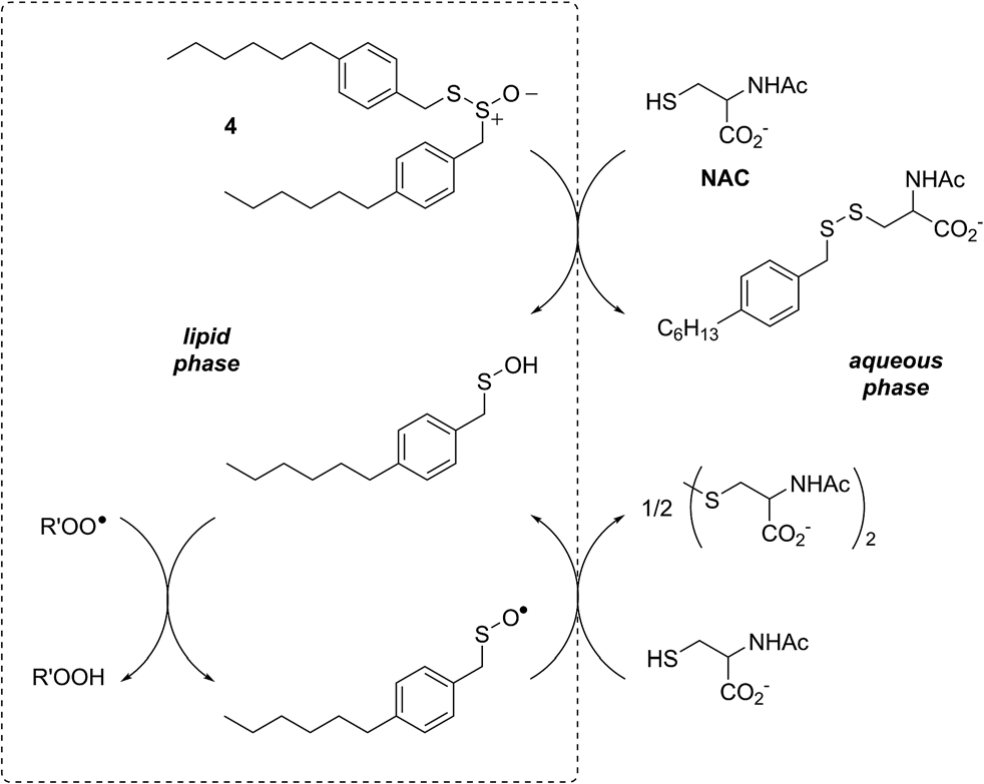
Proposed mechanism for the RTA activity of **4** observed in the presence of *N*-acetylcysteine (NAC).

In a preliminary communication, we reported that allicin and petivericin are not particularly effective RTAs in lipid bilayers.^37^ Surmising that this was because the sulfenic acids derived therefrom partitioned to the aqueous phase and underwent other reactions, we synthesized a lipophilic analog of petivericin (**4**), which we found to be an excellent RTA in lipid bilayers, *but only in the presence of a hydrophilic thiol* – conditions that did not improve the poor reactivity of allicin and petivericin.^37^ To account for these observations, the mechanism shown in Scheme 1 was proposed, where *S*-thiolation of **4** by *N*-acetylcysteine (NAC) produces a lipophilic sulfenic acid which traps lipophilic peroxyl radicals and can be regenerated *via* reaction with another molecule of NAC at the bilayer interface. Herein we report the full details of these preliminary studies, and have in the interim expanded the scope of our investigations to: (1) elucidate the structural factors that contribute to the efficacy of hexylated petivericin as a RTA in lipid bilayers, (2) provide experimental support for our proposed mechanism, and (3) include a comparative study of the antioxidant activity and cytotoxicity of allicin, petivericin and hexylated petivericin in mammalian cell culture. Taken together, the results reveal that allicin and petivericin are not RTAs in cells, but instead deplete glutathione and induce cell death. In contrast, hexylated petivericin appears to be effective in cells; its differing behavior likely being due to its greater partitioning to the lipid bilayer.

## Results

### I. Synthesis

**Scheme 2:**
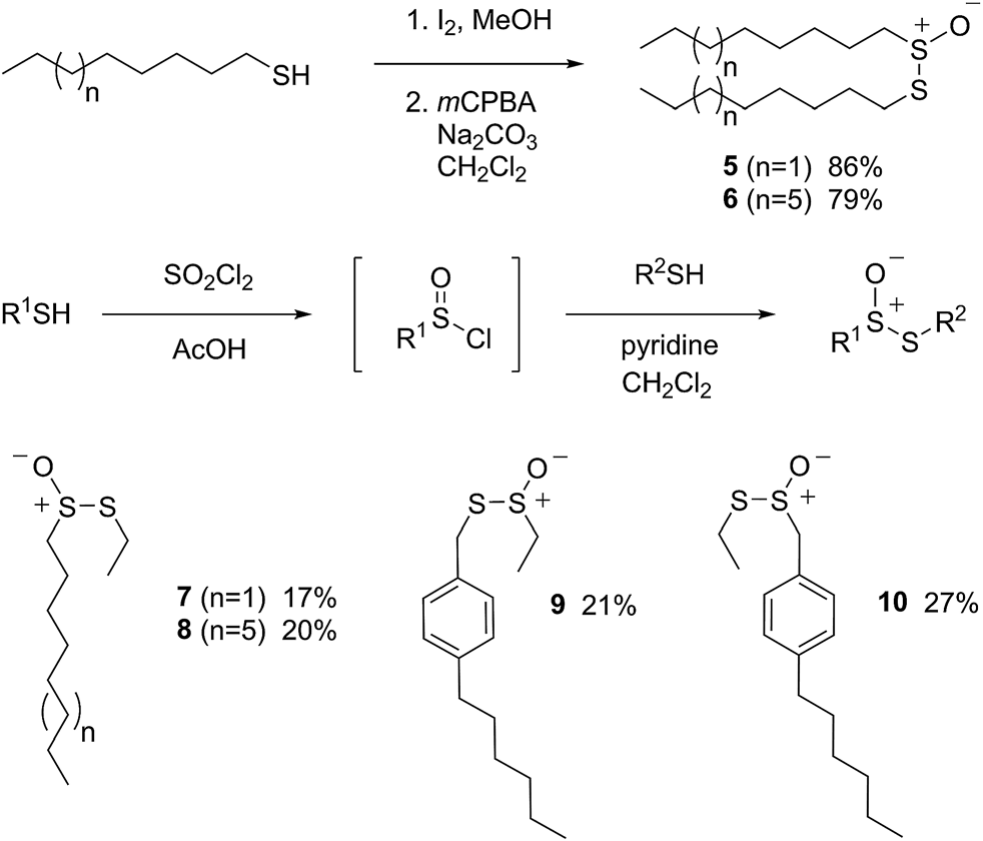
The preparation of thiosulfinates **5–10**.

Allicin (**1**),^33^ petivericin (**2**),^34^ 9-triptycenesulfenic acid (**3**)^35^ and hexylated petivericin (**4**)^37^ were prepared as described previously. The symmetrical *n*-alkylthiosulfinates **5** and **6** were prepared from the corresponding commercially available thiols *via* oxidation with I_2_ (5% solution in methanol), followed by oxidation with one equivalent of MCPBA, as shown in Scheme 2. The unsymmetrical thiosulfinates **7–10** were prepared by treatment of a thiol with sulfuryl chloride in acetic acid,^38^ followed by the addition of the second thiol to the resultant sulfinyl chloride as shown in Scheme 2. Complete details are given in the Experimental section.

### II. Phosphatidylcholine liposome oxidations – competition with H_2_B-PMHC

The RTA activities of allicin (**1**), petivericin (**2**), 9-triptycenesulfenic acid (**3**) and the synthetic thiosulfinates (**4–10**) were determined in phosphatidylcholine liposomes using H_2_B-PMHC, a fluorogenic lipid oxidation probe.^39^ In its unreacted state, the fluorescence of the BODIPY moiety of H_2_B-PMHC is quenched by electron transfer from the PMHC moiety (so-named after 2,2,5,7,8-pentamethyl-6-hydroxychroman, a truncated form of α-TOH). Upon reaction with peroxyl radicals the PMHC moiety is no longer sufficiently electron-rich to quench the BODIPY (eqn (3)), leading to a rapid rise in its fluorescence.^40^,^41^
3
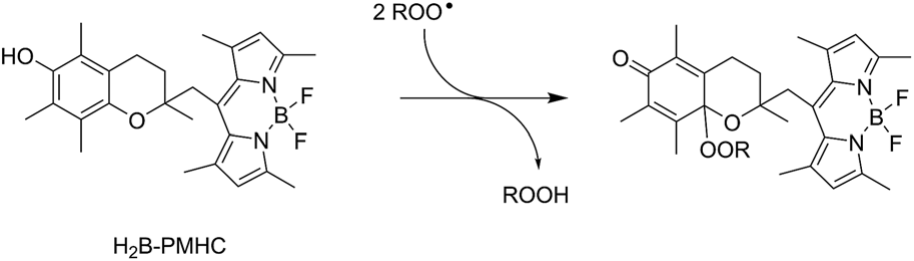



**Fig. 1:**
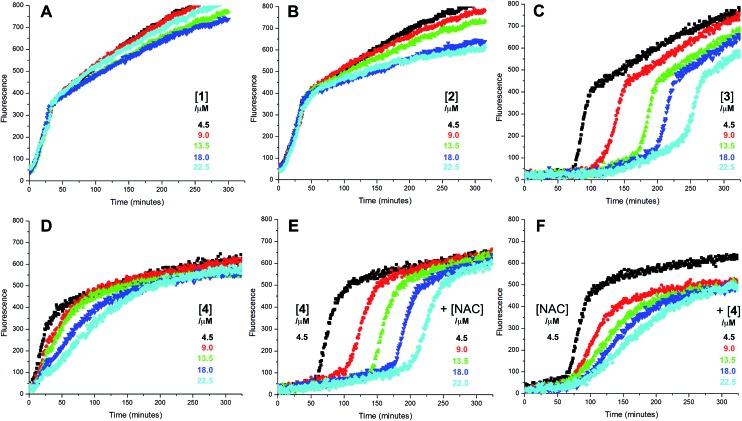
Representative fluorescence (at 520 nm) intensity–time profiles from MeOAMVN-mediated (0.2 mM) oxidations of phosphatidylcholine liposomes (1 mM in phosphate-buffered saline, pH 7.4) containing H_2_B-PMHC (0.15 μM) and increasing concentrations (4.5, 9.0, 13.5, 18 and 22.5 μM) of allicin (**1**, A), petivericin (**2**, B), 9-triptycenesulfenic acid (**3**, C) and hexylated petivericin (**4**, D) at 37 °C. Also shown are corresponding oxidations inhibited by 4.5 μM of **4** and increasing concentrations of *N*-acetylcysteine (1–5 equivalents) (E) and 4.5 μM *N*-acetylcysteine with increasing concentrations of **4** (1–5 equivalents) (F).

**Fig. 2:**
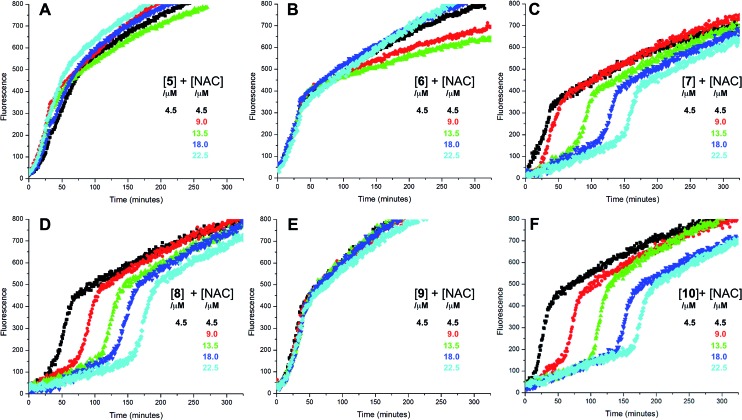
Representative fluorescence (at 520 nm) intensity–time profiles from MeOAMVN-mediated (0.2 mM) oxidations of phosphatidylcholine liposomes (1 mM in phosphate-buffered saline, pH 7.4) containing H_2_B-PMHC (0.15 μM) with 4.5 μM of either the symmetric *n*-alkylthiosulfinates **5** (A) and **6** (B), the unsymmetric *n*-alkylthiosulfinates **7** (C) and **8** (D) and hexylayted petivericin hybrids **9** (E) and **10** (F) with increasing concentrations of *N*-acetylcysteine (1–5 equivalents) at 37 °C.

Representative results are shown in Fig. 1 for oxidations initiated with the lipophilic azo initiator MeOAMVN at 37 °C.^42^ The initial rate of fluorescence increase is indicative of the relative rates of reaction of H_2_B-PMHC and the added antioxidant with peroxyl radicals.^70^ No inhibition of the oxidation of H_2_B-PMHC is observed in the presence of either **1** (Fig. 1A) or **2** (Fig. 1B) over the concentration range examined (4.5–22.5 μM). In contrast, the persistent sulfenic acid (**3**) was an excellent inhibitor (Fig. 1C). The expression in eqn (4), which is derived from a kinetic analysis of the initial rates of H_2_B-PMHC oxidation in the presence and absence of added RTA,^39^ enables derivation of the relative inhibition rate constants (hereafter referred to as *k*_rel_) from a plot of ln[(*I*_∞_ – *I*_*t*_)/(*I*_∞_ – *I*_0_)] *vs.* ln(1 – *t*/*τ*):4
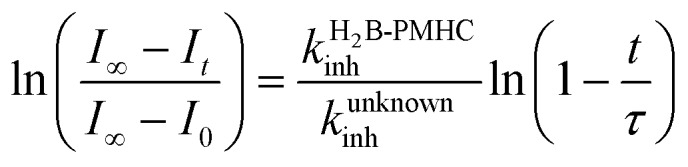



This analysis indicates that the rate constant for the reaction of **3** with lipophilic peroxyl radicals is a factor of 25 ± 3 greater than that of H_2_B-PMHC, which is known to react at roughly the same rate as α-TOH.^39^ The time required to reach maximum fluorescence in the first phase of the plots (*ca.* 400 counts), hereafter referred to as the ‘inhibited period’ (*τ* in eqn (4)), reflects the stoichiometry of the reaction of the added antioxidant and the peroxyl radicals. The inhibited periods in Fig. 1C correlate nicely with the concentration of **3** yielding a slope of 12 ± 0.8 min μM^–1^. Since the dependence of the inhibited period on the concentration of α-TOH under similar conditions is roughly twice that of **3**, and α-TOH is known to trap two peroxyl radicals,^43^ each molecule of **3** must trap only one MeOAMVN-derived peroxyl radical. A stoichiometry of one is consistent with the reactivity of compounds **1** and **2** in homogenous organic solution.^33^,^34^ Thiosulfinate **4**, a more lipophilic analog of petivericin (**2**), was able to retard the oxidation of H_2_B-PMHC (Fig. 1D), but did not display a clear inhibited period as was observed for the persistent sulfenic acid. Therefore, although **4** is clearly more reactive than **1** or **2**, it is far less reactive than the authentic sulfenic acid **3**.

Sulfenic acids are also formed from thiosulfinates by reaction with thiols. Therefore, we investigated the addition of a thiol to the liposome oxidations in the presence of **1**, **2** and **4**. We chose the popular glutathione analog *N*-acetylcysteine (NAC) as a model thiol for these studies. While the addition of NAC did not impact the rate of oxidation of H_2_B-PMHC in the presence of **1** or **2** (or when used alone, see ESI†), it had a marked effect when used in combination with **4** (Fig. 1E). The initial rates indicate an apparent *k*_rel_ of 9 ± 2 and the inhibited period scales with total antioxidant concentration (*i.e.* [**4**] + [NAC]) at 7.6 ± 0.2 min μM^–1^. Interestingly, when increasing concentrations of **4** are used with a constant concentration of NAC (Fig. 1F), the data appears to be the additive result of the first data set in Fig. 1E with the data in Fig. 1D. That is, there is a short inhibited period followed by a retarded phase.

Since each of **1**, **2** and **4** possess a similar pseudo-symmetric core structure with activated methylene groups adjacent the thiosulfinate moiety, the reactivities of six additional synthetic thiosulfinates were determined under the same conditions in an attempt to clarify any structure–reactivity relationships. Although the thiosulfinates **5–10** did not display any RTA activity in the absence of NAC (data not shown), significant activity was observed for some of these compounds in the presence of NAC. Representative results are shown in Fig. 2.

The lipophilic bis(*n-*alkyl)thiosulfinates **5** and **6** were ineffective when co-administered with increasing amounts of NAC. However, the unsymmetrical thiosulfinates **7** and **8** (wherein a ethyl group was substituted for one of the two octyl or dodecyl chains in **5** and **6**, respectively) were effective (*cf.*Fig. 2C and D). Although their reactivity was noticeably lower than for **4**, with apparent *k*_rel_ of 3.5 ± 0.9 and 4.5 ± 1.0, respectively, (compared to 9 ± 2 for **4**) the dependence of their inhibited periods on total antioxidant concentration (7.3 ± 0.4 and 6.6 ± 0.5 min μM^–1^, respectively), was essentially identical to **4** (7.6 ± 0.2 min μM^–1^). The two additional unsymmetrical thiosulfinates **9** and **10**, which differ only in the sulfur atom to which oxygen is attached, differed markedly in their reactivity. Thiosulfinate **10**, which is expected to react with NAC to yield the same sulfenic acid as that which arises in the *S*-thiolation of **4**, displays similar activity, but with a lower relative apparent rate constant of 3.6 ± 0.8. In sharp contrast, thiosulfinate **9** is devoid of any radical-trapping activity.

**Fig. 3:**
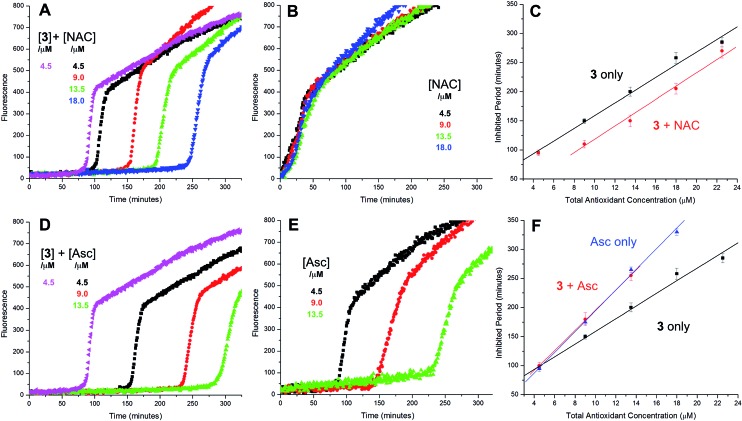
Representative fluorescence (at 520 nm) intensity–time profiles from MeOAMVN-mediated (0.2 mM) oxidations of phosphatidylcholine liposomes (1 mM in phosphate-buffered saline, pH 7.4) containing H_2_B-PMHC (0.15 μM) and 9-triptycenesulfenic acid (**3**, 4.5 μM) with increasing concentrations (1–5 equivalents) of NAC (A) or ascorbate (Asc, D) at 37 °C. Also shown are corresponding results for NAC (B) or ascorbate used alone (E). The inhibited periods are plotted as a function of total antioxidant concentration in panels C ([**3**] + [NAC]) and F ([**3**] + [ascorbate]).

The putative interaction between NAC and sulfenic acids in phosphatidylcholine liposomes was probed using the persistent 9-triptycenesulfenic acid as shown in Fig. 3A. In these experiments, NAC extended the inhibited period attributed to the sulfenic acid while maintaining the same radical-trapping kinetics, despite not being able to inhibit the oxidation of H_2_B-PMHC on its own (Fig. 3B). Moreover, the dependence of the inhibited period on the total antioxidant concentration (sulfenic acid and NAC) of 12 ± 0.6 min μM^–1^ is indistinguishable from that of the sulfenic acid alone (12 ± 0.8 min μM^–1^, data in Fig. 1C; see comparison in Fig. 3C). Ascorbate was also used in conjunction with the sulfenic acid in place of NAC (Fig. 3D). Similar to NAC, ascorbate also extends the inhibition period attributed to the sulfenic acid. With ascorbate, the dependence of the length of the inhibited period on the total antioxidant concentration (sulfenic acid and ascorbate) is slightly larger (15 ± 0.9 min μM^–1^) than the sulfenic acid alone (12 ± 0.8 min μM^–1^, data in Fig. 1C; see comparison in Fig. 3F). From control experiments it appears that ascorbate inhibits oxidation (Fig. 3E), in contrast to NAC (Fig. 3B). However, it is likely that this apparent inhibition is in fact due to the reduction of the phenoxyl radical derived from H_2_B-PMHC oxidation,^43^ consistent with the known chemistry of ascorbate and PMHC or α-tocopherol.^44^

**Fig. 4:**
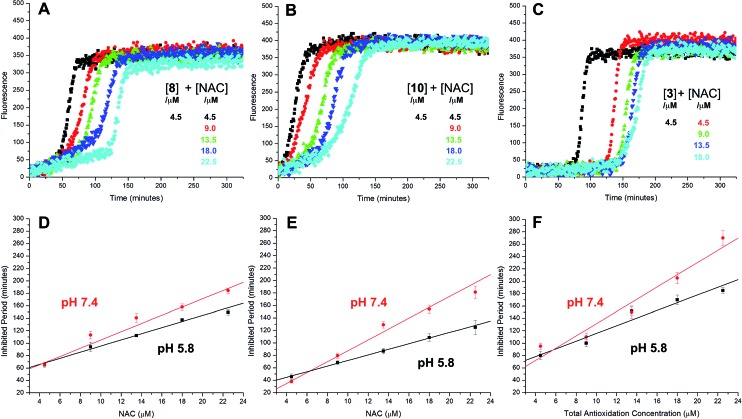
Representative fluorescence (at 520 nm) intensity–time profiles from MeOAMVN-mediated (0.2 mM) oxidations of phosphatidylcholine liposomes (1 mM in phosphate-buffered saline, pH 5.8) containing 0.15 μM H_2_B-PMHC, 4.5 μM of either **9** (A), **10** (B) or **3** (C) and increasing concentrations of NAC (1–5 equivalents) at 37 °C. Panels D–F show the dependence of the inhibited periods *versus* antioxidant concentration at pH 5.8 and 7.4.

The radical-trapping activity of a select number of thiosulfinates was also explored in buffer at acidic pH (5.8). Representative data are presented for thiosulfinates **8** and **10** in Fig. 4. A qualitative comparison of Fig. 4A and 2D suggests that while the *k*_rel_ of **8** does not change significantly at pH 5.8 relative to 7.4, the inhibited periods are noticeably shorter at lower pH. The same trend is evident on comparing the data for **10** in Fig. 4B and 2F. The inhibited periods are given as a function of added NAC at pH 5.8 and 7.4 for **8** and **10** in Fig. 4D and E, respectively. For comparison, analogous data were obtained in the presence of the persistent sulfenic acid **3**, and is shown in Fig. 4C and F.

### III. PLPC liposome oxidations – determination of hydroperoxides

Lipid hydroperoxide formation was also monitored directly in a select set of MeOAMVN-mediated oxidations of liposomes (at 37 °C) made exclusively from a polyunsaturated phospholipid (1-palmitoyl-2-linoleyl-*sn*-glycero-3-phospho-choline, PLPC). The hydroperoxide concentrations were determined using a phosphine–coumarin conjugate, which undergoes fluorescence enhancement upon oxidation to the phosphine oxide in the presence of hydroperoxides (eqn (5)).^45^ This probe has been shown to provide hydroperoxide concentrations in inhibited hydrocarbon autoxidations that are indistinguishable from those derived using conventional methods.^45^5
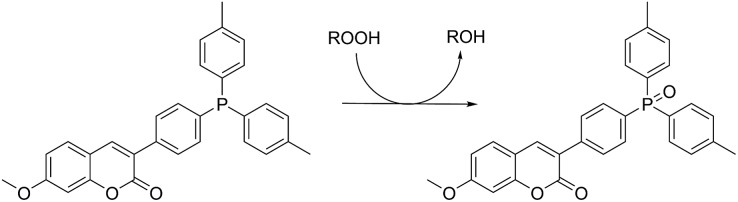



**Fig. 5:**
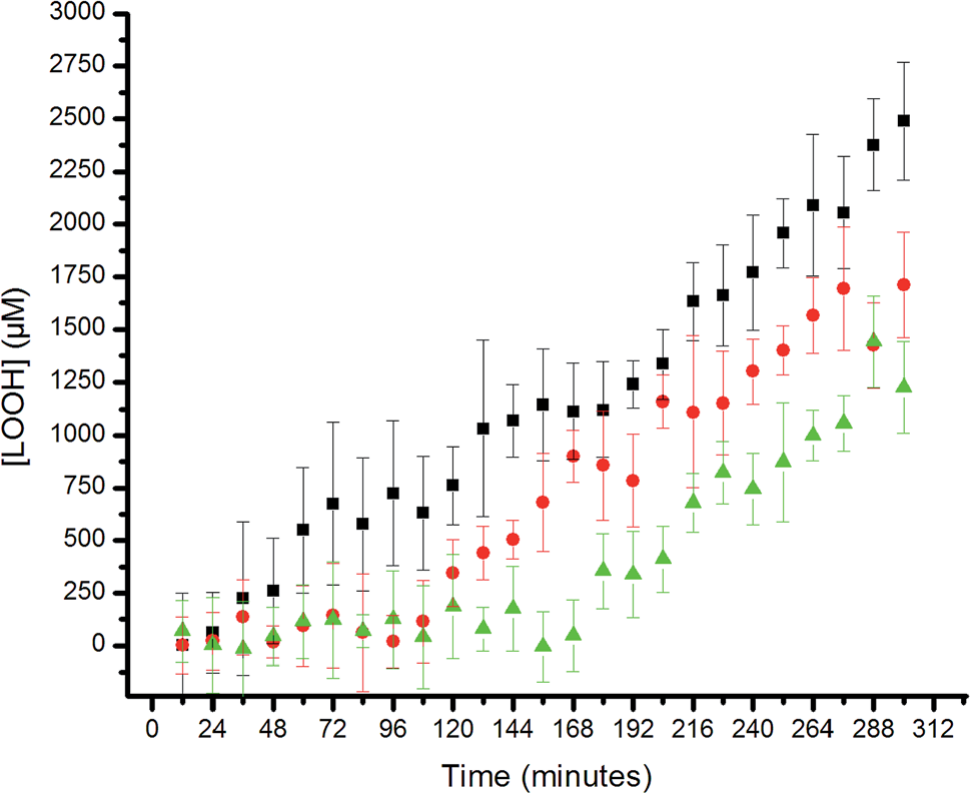
Hydroperoxide formation in the autoxidation of 1-palmitoyl-2-linoleyl-*sn-glycero*-3-phosphocholine liposomes (13.3 mM in phosphate-buffered saline, pH 7.4) initiated by MeOAMVN (150 μM) at 37 °C in the presence of 25 μM **4** + 25 μM *N*-acetylcysteine (circles), 25 μM **4** + 50 μM *N*-acetylcysteine (triangles) or no additives (squares).

The results are shown in Fig. 5. The uninhibited autoxidation displays a linear growth in [ROOH] with time, as expected for the initial part of the reaction (<20% conversion). Addition of 25 μM of each of the hexylated petivericin and NAC afforded a clear inhibited period, where ROOH production is effectively suppressed for *ca.* 96 minutes. When two equivalents of NAC are used with hexylated petivericin, the inhibited period is extended further, to roughly 170 minutes. In contrast, hexylated petivericin or NAC alone did not suppress lipid peroxidation (see ESI†).

### IV. Inhibition of lipid peroxidation and cytotoxicity in cell culture

**Fig. 6:**
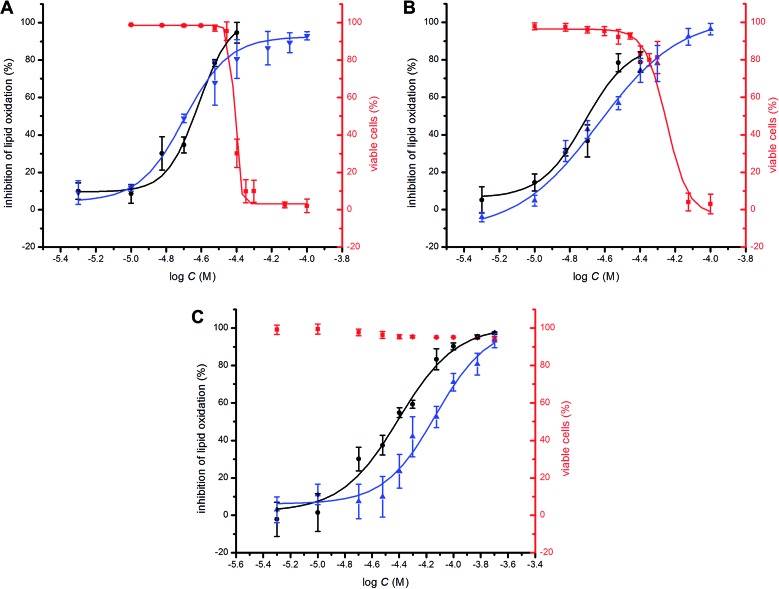
Representative dose–response curves obtained from flow cytometry (1 × 10^6^ cells mL^–1^; *λ*_ex_ = 488 nm, *λ*_em_ = 525 ± 25 nm; 10 000 events) following induction of oxidative stress by addition of either diethylmaleate (9 mM) or RSL3 (4 μM) in TF1a cells grown in RPMI media containing either allicin (A), petivericin (B) or hexylated petivericin (C) for 22 hours at 37 °C. The lipid peroxidation reporter C11-BODIPY^581/591^ (1 μM) was added to each of the cell cultures for 30 minutes prior to either DEM (blue) or RSL3 (black) treatment. Cell viability (red) was also determined by flow cytometry (5 × 10^5^ cells mL^–1^; *λ*_ex_ = 488 nm, *λ*_em_ = 675 ± 25 nm; 10 000 events) following treatment of the cells pre-incubated with allicin (A), petivericin (B) or hexylated petivericin (C) for 22 hours at 37 °C with a solution of 7-aminoactinomycin D (5 μL/1 × 10^5^ cells, 10 min). See the ESI† for analogous dose–response curves in HEK-293 cells.

To probe the potential biological activity of hexylated petivericin, its efficacy in preventing lipid peroxidation was determined in human TF1a erythroblasts and HEK-293 embryonic kidney cells and compared to allicin and petivericin. The lipophilic C11-BODIPY^581/591^ probe was used to monitor membrane lipid oxidation;^46^ it is oxidized competitively with unsaturated membrane lipids and undergoes significant enhancement in its fluorescence at *ca.* 520 nm upon oxidation. Lipid peroxidation was initiated either by depleting cells of glutathione with diethylmaleate (DEM) or by inhibition of glutathione peroxidase-4 (Gpx4) with RSL3.^47^ Representative dose–response curves for experiments with TF1a cells are shown in Fig. 6 alongside cytotoxicity studies carried out in parallel using the 7-aminoactinomycin (7-AAD) fluorophore.

A clear dose-dependence was observed when each of allicin (Fig. 6A), petivericin (Fig. 6B) and hexylated petivericin (Fig. 6C) were used to inhibit lipid peroxidation, with EC_50_ values of 20 ± 1, 23 ± 2 and 74 ± 9 μM, respectively, when DEM was used as the initiator. Similar trends were observed in HEK-293 cells (see ESI†). Although the EC_50_ values were essentially the same when either allicin or petivericin were used to inhibit lipid peroxidation initiated with RSL3 (EC_50_ = 24 ± 2 and 19 ± 2 μM, respectively), hexylated petivericin seemed relatively more potent (EC_50_ = 39 ± 3 μM). Allicin and petivericin induced cell death at concentrations similar to those that were effective in inhibiting lipid peroxidation, *i.e.* TC_50_ = 39 ± 1 and 56 ± 3 μM, respectively, (in TF1a erythroblasts; similar trends were observed in HEK-293 cells, see ESI†), while hexylated petivericin had no impact on cell viability throughout the concentration range studied (5–200 μM).

### V. Effect of thiosulfinates on cellular thiol concentration

**Fig. 7:**
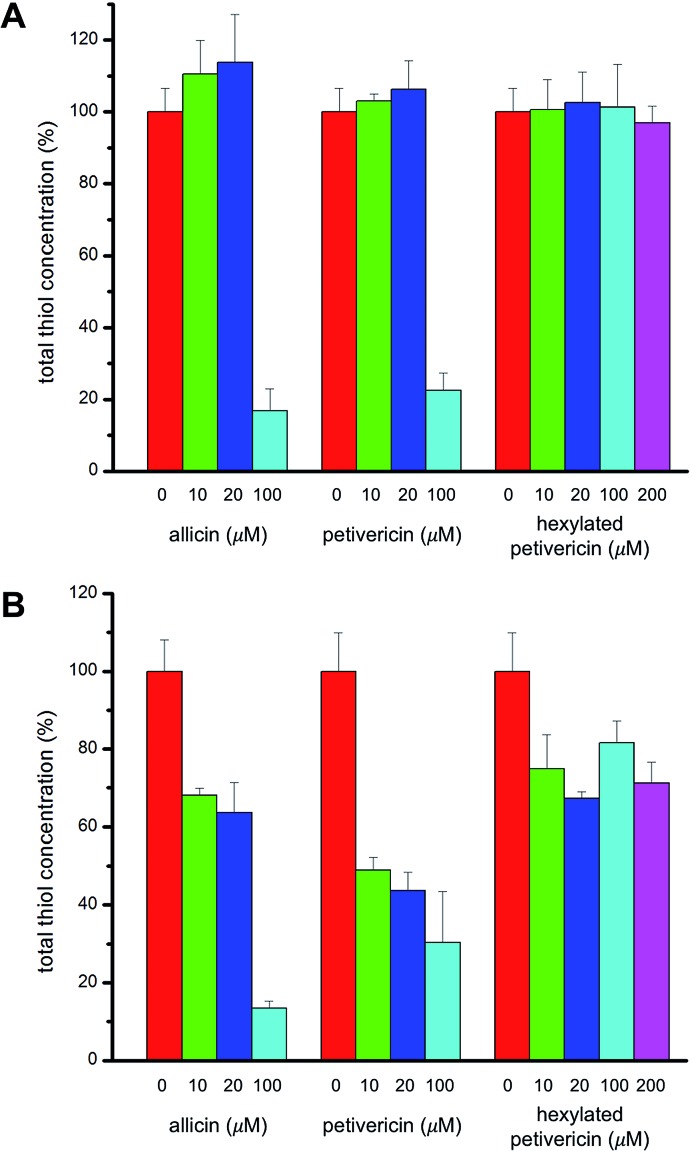
Cellular thiol concentration determined as a function of thiosulfinate concentration after 22 hours incubation in either TF1a (A) or HEK-293 cells (B). Thiol concentrations were determined relative to total protein in a minimum of three trials.

Given allicin's proclivity to react with glutathione and other cellular thiols,^68^ we determined the effect of each of allicin, petivericin and hexylated petivericin on total cellular thiols over the concentration range employed in the foregoing lipid peroxidation/cytotoxicity assays. Following incubation of cells in media supplemented with varying amounts of each thiosulfinate for 22 hours, cells were lysed and the protein concentration and total thiol concentration were determined. Small increases were observed for allicin at non-lethal concentrations in TF1a cells (*e.g.* 10 and 20 μM, *cf.*Fig. 7). The trend persists, but is even less obvious for petivericin, and thiol levels were severely reduced relative to total protein at cytotoxic concentrations of both compounds. However, hexylated petivericin, showed essentially no effect on total thiol concentration over the concentration range studied. Interestingly, a different profile was observed for allicin and petivericin in HEK-293 cells, where thiol was progressively depleted. In contrast, and consistent with the results in TF1a cells, progressive diminution in cellular thiol with increasing concentration of hexylated petivericin was not observed in HEK-293 cells.

### VI. Electrophilic potential of allicin, petivericin and hexylated petivericin

To demonstrate that each of allicin, petivericin and hexylated petivericin have similar inherent reactivity toward cellular thiols, we measured their potency as inhibitors of papain, the archetype cysteine protease. Papain was incubated with each thiosulfinate and its protease activity was then determined using a chromogenic substrate. The data (see ESI† for dose–response curves) reveal essentially identical IC_50_ values for each of allicin (1.2 ± 0.2 μM), petivericin (1.1 ± 0.1 μM) and hexylated petivericin (1.0 ± 0.1 μM).

## Discussion

Allicin and petivericin are effective RTAs in homogenous organic solution because they undergo Cope elimination to yield 2-propenesulfenic acid and phenylmethanesulfenic acid, respectively, and these molecules react with peroxyl radicals without an enthalpic barrier.^33^,^34^ The foregoing experiments were undertaken to assess the RTA activity of allicin and petivericin in more biologically relevant contexts: the lipid bilayers of liposomes and mammalian cells. In liposomes, allicin and petivericin were found to be at least 150 times less reactive than α-TOH, while an authentic sulfenic acid, 9-triptycenesulfenic acid, was roughly 25-times more reactive than α-TOH. Reconciling these results requires that either: (1) Cope elimination of 2-propenesulfenic acid and phenylmethanesulfenic acid from allicin and petivericin, respectively, is slowed in the lipid bilayer due to H-bonding at the interface,^71^ or (2) the sulfenic acids produced are sufficiently hydrophilic to partition to the aqueous phase where they undergo other reactions (*i.e.* oxidation and condensation).^72^ The former explanation can be ruled out since the kinetics of allicin and petivericin decomposition (due to Cope elimination) was similar in liposomes (see ESI†) and homogenous organic solution.^33^,^34^ The latter explanation is supported by the significantly higher reactivity observed for thiosulfinate **4**, an analog of petivericin which includes *n*-hexyl substitution at the 4-position of the phenyl ring. The requirement for Cope elimination of (4-hexylphenyl)methanesulfenic acid to account for the activity of **4** is evident in its diminished kinetics for radical-trapping (*k*_rel_ = 0.9 ± 0.1) relative to the persistent sulfenic acid **3** (*k*_rel_ = 25 ± 3); a difference which is fully consistent with that observed in chlorobenzene, where petivericin and **3** have *k*_inh_ values of 2.0 × 10^5^ and 30 × 10^5^ M^–1^ s^–1^, respectively.^34^,^36^

**Scheme 3:**
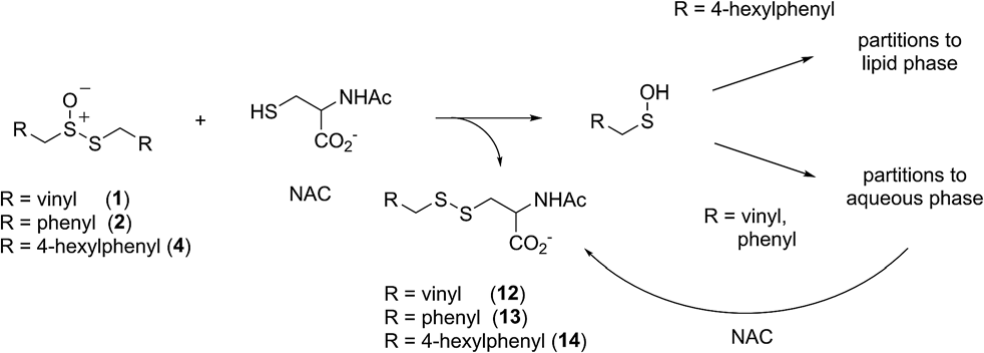
Allicin and petivericin undergo *S*-thiolation by NAC to yield sulfenic acids that partition to the aqueous phase where they can react with NAC, whereas hexylated petivericin undergoes *S*-thiolation to yield a lipophilic sulfenic acid.

Sulfenic acids are also formed from thiosulfinates by *S*-thiolation reactions – with either a thiol or another molecule of thiosulfinate as the nucleophile.^1^,^2^,^4^ Since thiols are ubiquitous *in vivo*, with some tissues containing mM concentrations of glutathione, it is plausible that such an interaction may contribute to any potential RTA activity of allicin in biological contexts. Interestingly, while the addition of *N*-acetylcysteine had no impact on the RTA activity of either allicin or petivericin in liposomes, there was a significant increase in activity of hexylated petivericin. Since allicin (and petivericin, see ESI†) are rapidly consumed in the presence of NAC, this implies that the hydrophilic sulfenic acids that are produced are consumed by reaction with another equivalent of thiol to give a disulfide. *S*-thiolation of hexylated petivericin, however, yields a lipophilic sulfenic acid that can trap lipophilic peroxyl radicals (Scheme 3).

**Scheme 4:**
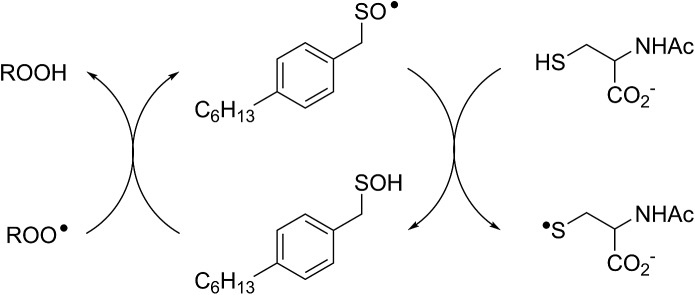
Regeneration of the lipophilic sulfenic acid derived from **4** (see Scheme 3) by NAC.

Interestingly, LC/MS analysis of aliquots from liposome oxidations inhibited by hexylated petivericin in the presence of NAC showed little (*ca.* 5%) conversion of the thiosulfinate to the corresponding mixed disulfide **14**. Nevertheless, the inhibited periods observed in Fig. 2 for the combination of hexylated petivericin and NAC are consistent with a radical-trapping capacity that exceeds the small amount of the lipophilic sulfenic acid that corresponds to the amount of **14** observed by LC/MS. Moreover, the addition of increasing amounts of NAC beyond one or two equivalents led to a concentration-dependent extension in the inhibited period, while maintaining the same overall kinetics – suggesting that NAC is able to regenerate the lipophilic sulfenic acid derived from **4** (Scheme 4).

The sequence in Scheme 4 is further supported by the results of liposome oxidations carried out in the presence of the persistent 9-triptycenesulfenic acid **3**. The addition of increasing amounts of NAC lead to a concentration-dependent extension in the inhibited period, while maintaining the same overall kinetics as when **3** alone was present.^74^ The dependence of the inhibited period on the concentration of NAC was essentially indistinguishable from the dependence of the inhibited period on the concentration of **3** alone, suggesting that the sulfenic acid was fully regenerated by NAC.^75^

The regeneration of a lipid-soluble RTA using a water-soluble reducing agent is well-precedented. The most famous example is the combination of α-TOH and ascorbate;^44^,^48^ ascorbate reduces the α-tocopheroxyl radical that is formed following the reaction of α-TOH with lipophilic peroxyl radicals, thereby effectively converting a water-soluble reducing equivalent into a lipid-soluble one. While thiols do not regenerate α-TOH from its corresponding α-tocopheroxyl radical,^49^,^50^ NAC is believed to regenerate simple selenophenols from their corresponding selenophenoxyl radicals when NAC is used in great excess.^50^,^51^ Since a direct H-atom transfer is thermodynamically unfavourable for the reaction of NAC with the selenophenoxyl radicals, it was suggested that the reaction occurs by electron transfer from the thiolate to the selenophenoxyl radical. The electron transfer would be rendered irreversible by protonation of the selenophenoxide and partitioning of the selenophenol to the organic phase, with concomitant formation of the NAC-derived disulfide. It would appear necessary to invoke such a mechanism for the regeneration of a sulfenic acid with NAC; direct H-atom is highly unfavourable on thermodynamic grounds (the RSO-H BDE is *ca.* 18 kcal mol^–1^ weaker than the RS-H BDE),^33^,^35^,^52^ but the redox couples for RSO˙/RSO^–^ and RSSR/RS^–^ are 0.74 (ref. 35) and –0.20 (ref. 53) V *vs.* NHE in acetonitrile and water (pH 7),^73^ respectively, suggest that the electron transfer is feasible.

Independent evidence that *S*-thiolation (and not Cope elimination) is responsible for the formation of (4-hexylphenyl)methanesulfenic acid from **4** in lipid bilayers comes from studies of thiosulfinates that lack an activated methylene group adjacent the divalent sulfur atom – necessary for facile Cope elimination.^34^ Interestingly, while the symmetrical bis(*n*-octyl)thiosulfinate and bis(*n*-dodecyl)thiosulfinate were ineffective, the corresponding unsymmetrical thiosulfinates wherein either the octyl or dodecyl chain adjacent the divalent sulfur was replaced with an ethyl group displayed reactivities similar to the hexylated petivericin. Since the sulfenic acid that results from *S*-thiolation would be identical from thiosulfinates **5** and **7** (or **6** and **8**), the lack of activity of **5** and **6** suggests that these compounds are too hydrophobic for efficient *S*-thiolation, which presumably takes place at the lipid/aqueous interface.

**Scheme 5:**
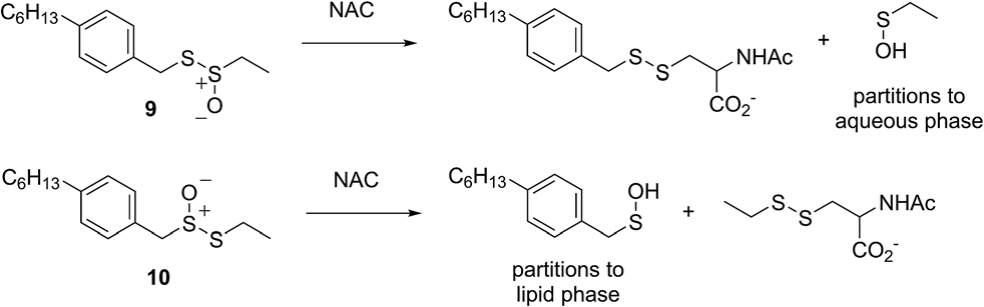
The disparate behaviour of thiosulfinates **9** and **10** is believed to result from the differing lipophilicities of the sulfenic acids formed by *S*-thiolation with NAC.

The importance of the lipophilicity of the sulfenic acid was demonstrated unequivocally by experiments with the unsymmetrical thiosulfinates **9** and **10**. Thiosulfinate **9**, which is expected to undergo *S*-thiolation with NAC to produce ethanesulfenic acid was completely ineffective, while thiosulfinate **10**, which is expected to undergo *S*-thiolation with NAC to yield (4-hexylphenyl)methanesulfenic acid, was similarly effective to hexylated petivericin. The disparate behaviour of **9** and **10** is illustrated in Scheme 5.

Comparison of the dependence of the inhibited periods as a function of added NAC suggests that the regeneration of the persistent sulfenic acid **3** (12 ± 0.6) is more efficient than the regeneration of the sulfenic acids derived from the thiosulfinates **4** (7.6 ± 0.2), **7** (7.3 ± 0.4), **8** (6.6 ± 0.5) and **10** (8.4 ± 0.6). This difference reflects the relative persistence of the sulfinyl radicals formed following H-atom transfer from the sulfenic acids to peroxyl radicals. The sulfinyl radical derived from **3** is known to be persistent under the experimental conditions due to steric hindrance,^35^ enabling it to be quantitatively reduced by NAC. In contrast, unhindered sulfinyl radicals are expected to be less persistent,^34^ and self-reactions as well as reactions with O_2_ and/or peroxyl radicals can compete with reduction by NAC. The pH dependence of the radical-trapping activities of the thiosulfinates further reinforces this point. While the apparent rates of radical-trapping do not vary significantly with pH, the dependence of the length of the inhibited period on antioxidant concentration decreases. This is consistent with slower regeneration of the sulfenic acid by the lower concentration of the thiolate form of NAC present at more acidic pH, allowing more time for deleterious side reactions.

Despite the numerous reports of allicin as a RTA (*vide supra*), no reports of cell-based assays of this activity can be found in the literature. Likewise for petivericin – although this is understandable since it was only relatively recently identified as the primary thiosulfinate in *Petiveria*.^54^ Allicin, petivericin and hexylated petivericin were able to prevent oxidation of the lipid peroxidation reporter C11-BODIPY^581/591^ in TF1a erythroblasts (and HEK-293 kidney cells, see ESI†), with EC_50_ values of 20 ± 1, 23 ± 2 and 74 ± 9 μM, respectively. In light of the lack of RTA activity displayed by allicin and petivericin in liposomes, at first glance these data suggest that the antioxidant mechanism of the three compounds cannot be due to radical-trapping.

Given the well known electrophilic reactivity of thiosulfinates, it has been suggested that allicin's antioxidant activity arises from upregulation of expression of phase II detoxifying enzymes. This leads to an increase in the cellular glutathione level, presumably *via* activation of the transcription factor Nrf2.^55^–^57^ In this connection, the trend in efficacies of the thiosulfinates at inhibiting lipid peroxidation in the TF1a (and HEK-293) cells may simply be due to their differing reactivity with nucleophilic cysteines on KEAP1. KEAP1 is the cytosolic protein which prevents Nrf2 translocation to the nucleus.^58^ Cysteine modification by electrophiles mediates KEAP1/Nrf2 signaling events, and is now believed to underlie the activities of most nutritional antioxidants.^59^ However, our own measurements indicate that allicin and petivericin do not upregulate GSH production to any significant extent in either TF1a or HEK-293 cells; rather they promote a visible decrease in GSH in the latter. Moreover, hexylated petivericin has no significant effect on GSH levels in either cell type.

Allicin's reactivity as an electrophile is apparently paradoxical; it is also believed to underlie its antimicrobial and anticancer activities. It has been determined to be highly cytotoxic to a wide variety of human cancer cells, including mammary MCF-7, endometrial and HT-29 colon cells,^60^ HeLa and SiHa cervical and SW480 colon cells,^61^ gastric epithelial cells,^62^ and leukemia HL60 and U937 cells,^63^ with EC_50_ values in the low micromolar range under similar conditions to those employed here. The antiproliferative activity of allicin has been ascribed to activation of the mitochondrial apoptotic pathway by GSH depletion and concomitant changes in the intracellular redox status. While petivericin has been reported to display antimicrobial and antifungal activities,^64^ there has been, to the best of our knowledge, no report on its toxicity to mammalian cells. Given the structural similarity between allicin and petivericin, it seems reasonable to suggest that it shares the same mechanisms.

Since the concentrations of allicin necessary to prevent lipid peroxidation in our assays have been reported to induce death in some human cell lines, we determined the cytotoxicities of allicin, petivericin and hexylated petivericin toward the same human TF1a and HEK-293 cells used in the lipid peroxidation assays. These studies revealed that allicin and petivericin induced cell death at concentrations only marginally higher than those necessary to inhibit lipid peroxidation (TC_50_ = 39 ± 1 and 56 ± 3 μM for allicin and petivericin, respectively). This suggests that the lipid peroxidation inhibition observed for allicin and petivericin may result simply from growth arrest and the resultant slowing (or halting) of aerobic metabolism associated with their toxicity. Accordingly, it would appear inappropriate to refer to allicin or petivericin as antioxidants in a biological context. In contrast, hexylated petivericin was not toxic throughout the concentration range examined in the lipid peroxidation assay (5–200 μM). This may be ascribed to its lipophilicity, which ensures localization to the lipid bilayer and thereby diminishes its reactivity with glutathione or nucleophilic cysteines on pro-apoptotic signaling proteins. The role of phase separation is supported by the lack of effect of hexylated petivericin on cellular thiol levels despite the results of proof-of-principle experiments wherein the three thiosulfinates were found to be equally potent inhibitors of papain, the archetype cysteine protease, when in homogenous solution.

As a result of its increased lipophilicity, rather than simply killing cells as does allicin and petivericin, hexylated petivericin appears to be a *bona fide* RTA. This difference is underscored by the results obtained when lipid peroxidation was induced by Gpx4 inhibition with RSL3 rather than GSH depletion with DEM; the relative efficacy of hexylated petivericin increased, while no change was observed for either allicin or petivericin. Unlike DEM administration, RSL3 inhibition of Gpx4 does not lead to a precipitous drop in GSH levels, leaving it available to recycle the lipophilic sulfenic acid thereby increasing the relative potency of hexylated petivericin.

## Conclusions

The garlic-derived thiosulfinate allicin and the analogous secondary metabolite from the related species *Petiveria alliacae*, do not trap peroxyl radicals in lipid bilayers. The sulfenic acids that derive from these thiosulfinates by either Cope elimination or *S*-thiolation are not sufficiently lipophilic to be retained in the lipid bilayer, precluding their reaction with peroxyl radicals. In contrast, synthetic thiosulfinates which give rise to lipophilic sulfenic acids are highly effective RTAs, provided that the thiosulfinates are sufficiently amphiphilic for *S*-thiolation to take place at the interface of the lipid and aqueous phases. Thiols serve not only to yield sulfenic acids *via S*-thiolation, but they are also capable of regenerating the sulfenic acids by reducing the sulfinyl radicals (formed following formal H-atom transfer to peroxyl radicals) by electron transfer from the corresponding thiolate. The results of experiments in human erythroblasts and embryonic kidney cells suggest that allicin and petivericin do not inhibit lipid peroxidation in cells, but induce cell death as a result of, or concomitant with, glutathione depletion. In contrast, hexylated petivericin is able to inhibit lipid peroxidation without inducing cell death or altering glutathione levels, presumably due to its more favourable partitioning to the lipid bilayer. The greater apparent activity of hexylated petivericin observed when lipid peroxidation is induced by Gpx4 inhibition with RSL3 suggests that the mechanism that operates in the lipid bilayers of liposomes extends to those that make up, and are found within, cells.

## Experimental section

### I. Materials

Egg phosphatidylcholine, penicillin–streptomycin, Dulbecco’s phosphate buffered saline (DPBS), phosphate buffered saline (PBS), MeOAMVN, *N*-acetylcysteine, ascorbate, trypan blue, C11-BODIPY^581/591^ (4,4-difluoro-5-(4-phenyl-1,3-butadienyl)-4-bora-3*a*,4*a*-diaza-*s*-indacene-3-undecanoic acid), RPMI-1640 media with/without phenol red, MEM media with/without phenol red, fetal bovine serum (FBS) and 7-aminoactinomycin D (7-AAD) were purchased from commercial sources and used as received. BCA protein assay kit was purchased from Thermo Scientific. Palmitoyl-2-linoleyl-*sn-glycero*-3-phosphocholine (PLPC) was synthesized according to the literature with purification by column chromatography immediately before use.^65^ H_2_B-PMHC and the hydroperoxide probe in eqn (5) were prepared as described in ref. 66 and 45, respectively. Allicin, petivericin, 9-triptycenesulfenic acid and hexylated petivericin was prepared as described in ref. 33–35 and 37, respectively. Thiosulfinates **5–10** were prepared as described below.

#### *S*-Octyl octane-1-sulfinothioate (**5**)

To a solution of octane-1-thiol (292 mg, 2 mmol) in MeOH (10 mL) at 0 °C, Iodine solution (5% in methanol) was added dropwise till the reddish color of iodine stayed, and then keeping the reaction stirring at 0 °C for another 20 min. Sodium thiosulfate was added to quench the excess iodine. The mixture was evaporated to remove the methanol mostly. The residue was treated with water (10 mL) and the resulting solution was extracted with ether. The extracts were combined, dried over magnesium sulphate and vacuumed to give a oil, which was dissolved in DCM (10 mL) at 0 °C. *m*-Chloroperbenzoic acid (*m*-CPBA) (77%, 470 mg, 2.10 mmol) in dichloromethane (5 mL) was added dropwise at 0 °C. The mixture was stirred at 0 °C for one hour. Sodium carbonate (2 g) was added in small portions with vigorous stirring. The reaction mixture was stirred for an additional 1 h at 0 °C. The reaction mixture was then filtered through magnesium sulfate. The filtrate was concentrated under reduced pressure yielding crude product, which was purified by flash chromatography on silica gel (hexane : ethyl acetate = 5 : 1) to give compound **5** as a clear oil (264 mg, 86% yield). ^1^H NMR (300 MHz, CDCl_3_): *δ* 3.06–3.20 (m, 4H), 1.73–1.87 (m, 4H), 1.27–1.51 (m, 20H), 0.85–0.89 (m, 6H); ^13^C NMR (75.5 MHz, CDCl_3_): *δ* 56.20, 32.87, 31.72, 31.68, 30.82, 29.08, 28.97, 28.57, 23.42, 22.59, 22.57, 14.05; HRMS (EI+) calcd for C_16_H_35_S_2_O [M + H]^+^ 307.2129, obsd 307.2135.

#### *S*-Dodecyl dodecane-1-sulfinothioate (**6**)

The title compound was obtained as for **5** and isolated as a white solid by recrystallization from ether (330 mg, 79%). ^1^H NMR (300 MHz, CDCl_3_): *δ* 3.06–3.20 (m, 4H), 1.73–1.87 (m, 4H), 1.27–1.51 (m, 20H), 0.85–0.89 (m, 6H); ^13^C NMR (75.5 MHz, CDCl_3_): *δ* 56.20, 32.87, 31.72, 31.68, 30.82, 29.08, 28.97, 28.57, 23.42, 22.59, 22.57, 14.05; HRMS (EI+) calcd for C_12_H_25_S [M – C_12_H_25_SO]^+^ 201.1677, obsd 201.1674; calcd for C_12_H_25_SO [M – C_12_H_25_S]^+^ 217.1626, obsd 217.1652.

#### *S*-Ethyl octane-1-sulfinothioate (**7**)

A well-stirred mixture of octane-1-thiol (1.8 mL, 25 mmol) and acetic acid (1.43 mL, 25 mmol) is cooled to –20 °C. Sulfuryl chloride (4.27 mL, 52.5 mmol) is added dropwise over a period of 20 min. Gas evolution is observed during the addition. Stirring is continued for 30 min at –20 °C, and then the mixture was allowed to warm up to room temperature over a period of 2 h and stirred at room temperature for 2 h. Acetyl chloride is vacuumed off to leave the sulfinyl chloride. To a solution of sulfinyl chloride (1.57 g, 8 mmol) in DCM (10 mL), pyridine (695 mg, 8.8 mmol) was added dropwise at 0 °C. The mixture was stirred at 0 °C for 10 min, to which ethanethiol (62 mg, 1 mmol) in DCM (1 mL) was added dropwise. After stirring at 0 °C at 1 h, water (10 mL) was added slowly to quench the reaction. The organic layer was separated and water phase was extracted with ether (15 mL × 6). All the organic phase was combined and washed by HCl (1 M, 15 mL × 2) and brine (20 mL). The organic solvent was then removed under reduced pressure. The resulting oil was purified by flash chromatography on silica gel (hexane : ethyl acetate = 3 : 2) to give compound **7** as a light yellow oil (38 mg, 17% yield). ^1^H NMR (300 MHz, CDCl_3_): *δ* 3.26–3.21 (m, 2H), 3.09 (q, *J* = 7.5 Hz, 2H), 1.89–1.79 (m, 2H), 1.39 (t, *J* = 7.5 Hz, 3H), 1.33–1.21 (m, 10H), 0.82 (t, *J* = 6.9 Hz, 3H); ^13^C NMR (75.5 MHz, CDCl_3_): *δ* 62.8, 31.6, 30.6, 28.9, 28.8, 27.9, 23.4, 22.5, 15.1, 14.0; HRMS (EI+) calcd for C_8_H_17_SO [M – C_2_H_6_S]^+^ 161.1000, obsd 161.1071.

#### *S*-Ethyl dodecane-1-sulfinothioate (**8**)

The title compound was obtained as for **7** following flash column chromatography on silica gel (hexane : ethyl acetate = 3 : 1) as a light yellow oil (55 mg, 20%). ^1^H NMR (300 MHz, CDCl_3_): *δ* 3.26–3.21 (m, 2H), 3.09 (q, *J* = 7.5 Hz, 2H), 1.89–1.79 (m, 2H), 1.39 (t, *J* = 7.5 Hz, 3H), 1.33–1.21 (m, 18H), 0.82 (t, *J* = 6.9 Hz, 3H); ^13^C NMR (75.5 MHz, CDCl_3_): *δ* 62.7, 31.8, 30.6, 29.5, 29.4, 29.2, 29.1, 28.9, 27.9, 23.4, 22.6, 15.1, 14.0; HRMS (EI+) calcd for C_12_H_25_SO [M – C_2_H_6_S]^+^ 217.1626, obsd 217.1530.

#### *S*-Ethyl-(4-hexylphenyl)methanesulfinothioate (**9**)

The title compound was obtained as for **7** following recrystallization from diethylether as a white solid (48 mg, 21%). ^1^H NMR (300 MHz, CDCl_3_): *δ* 7.20–7.38 (m, 4H), 4.48 (t, *J* = 9.6 Hz, 2H), 2.76 (q, *J* = 7.5 Hz, 2H), 2.62 (t, *J* = 7.5 Hz, 2H), 1.65–1.55 (m, 2H), 1.35–1.26 (m, 6H), 1.20 (t, *J* = 7.5 Hz, 3H), 0.88 (t, *J* = 7.5 Hz, 3H); ^13^C NMR (75.5 MHz, CDCl_3_): *δ* 144.4, 131.2, 128.9, 124.9, 68.7, 35.6, 31.6, 31.2, 31.0, 28.8, 22.5, 14.8, 14.0; HRMS (EI+) calcd for C_2_H_5_S_2_O [M – C_13_H_19_]^+^ 108.9782, obsd 108.9764, calcd for C_13_H_19_ [M – C_2_H_5_S_2_O]^+^ 175.1487, obsd 175.1507.

#### *S*-4-Hexylbenzyl ethanesulfinothioate (**10**)

The title compound was obtained as for **7** following flash column chromatography on silica gel (hexane : ethyl acetate = 3 : 1) as a white solid (37 mg, 27%). ^1^H NMR (300 MHz, CDCl_3_): *δ* 7.14–7.29 (m, 4H), 4.31 (s, 2H), 2.93 (q, *J* = 7.5 Hz, 2H), 2.59 (t, *J* = 7.8 Hz, 2H), 1.65–1.54 (m, 2H), 1.33–1.26 (m, 9H), 0.88 (t, *J* = 7.8 Hz, 3H); ^13^C NMR (75.5 MHz, CDCl_3_): *δ* 143.2, 132.2, 129.0, 128.9, 57.2, 40.3, 35.6, 31.6, 31.3, 28.8, 22.6, 14.0, 8.1; HRMS (EI+) calcd for C_13_H_18_S [M – C_2_H_5_SOH]^+^ 206.1129, obsd 206.1107.

### II. Liposome preparation

Egg phosphatidylcholine (Egg PC) or PLPC (75 mg) was weighed in a dry vial and dissolved in a minimum volume of chloroform. The solvent was then evaporated under argon to yield a thin film on the vial wall. The film was left under vacuum for 2 hours to remove any remaining solvent. The lipid film was then hydrated with 4.0 mL of a 10 mM phosphate-buffered saline (PBS) solution (pH 7.4 or 5.8) containing 150 mM NaCl, yielding a 24 mM lipid suspension. The lipid suspension was then subjected to 10 freeze-thaw-sonication cycles, followed by extrusion using a mini-extruder equipped with a 100 nm polycarbonate membrane.

### III. Inhibited autoxidation of unilamellar PC liposomes

To individual 45 μL aliquots of the 24 mM liposome solution were added increasing amounts (4.5, 9, 13.5, 18 and 22.5 μL, respectively) of a solution of the test antioxidant in acetonitrile (0.15 mM) and 10 μL of a solution of H_2_B-PMHC in acetonitrile (13 μM). Each resultant solution was then diluted to 1 mL with PBS, from which 280 μL of each was loaded into a well of a 96-well microplate. The solution was equilibrated to 37 °C for 5 min, after which 20 μL of a solution of 3 mM in 2,2′-azobis-(4-methoxy-2,4-dimethylvaleronitrile) (MeOAMVN) in acetonitrile was added to each well using the reagent dispenser of a microplate reader. The fluorescence was then monitored for 6 h at 50 s time intervals (*λ*_ex_ = 485 nm; *λ*_em_ = 520 nm). The final solutions in each well were 1 mM in lipids, 0.15 μM in H_2_B-PMHC, 0.2 mM in MeOAMVN and either 4.5, 9, 13.5, 18, 22.5 μM in antioxidant. Each liposome contained, on average, 15 fluorophores with an Egg PC/fluorophore molar ratio of 6700 : 1. Under these conditions, no fluorescence self-quenching occurs within the liposome bilayer.

### IV. Inhibited autoxidation of unilamellar PLPC liposomes

Stock solutions of the different antioxidants in acetonitrile were added into various amounts of unilamellar PLPC liposomes in pH 7.4 buffer. MeOAMVN (48 μL of a 2.3 mM solution in acetonitrile) was then added to initiate lipid peroxidation. The reaction mixtures were stirred at 37 °C in an aluminium heating block. The final concentrations of PLPC and MeOAMVN were 8.75 mM and 0.15 mM, respectively. Every 12 minutes, 10 μL of the reaction mixture was withdrawn and transferred to a well of a 96-well microplate and 165 μL of MeOH containing butylated hydroxytoluene (45 mM) was added to destroy the liposome and prevent adventitious oxidation. Using the reagent dispenser of the microplate reader, 25 μL of a solution of **11** in acetonitrile (160 mM) was added to each well and the initial rate of the reaction was obtained by measuring the fluorescence (*λ*_ex_ = 340 nm; *λ*_em_ = 425 nm) for 50 s using a microplate reader. The lipid hydroperoxide concentration was calculated based on the initial rate of the reaction.^45^

### V. Cellular lipid peroxidation

TF1a cells were cultured in RPMI-1640 media with 10% FBS and 1% penicillin–streptomycin. HEK-293 cells were cultured in MEM media with 10% FBS, 1% non-essential amino acid, 1 mM sodium pyruvate and 1% penicillin–streptomycin. Cells (5 × 10^5^ cells mL^–1^ or 1 × 10^6^ cells mL^–1^) were treated with each of the thiosulfinates **1**, **2** and **4** at final concentrations from 5 μM to 200 μM and incubated at 37 °C for 22 hours in phenol red-free RPMI-1640 media with 10% FBS and 1% penicillin–streptomycin for TF1a cells and phenol red-free MEM media with 10% FBS, 1% non-essential amino acid, 1 mM sodium pyruvate and 1% penicillin–streptomycin for HEK-293 cells in a humidified atmosphere containing 5% CO_2_ in air. Cells were then treated with 1 μM C11-BODIPY^581/591^ in media and incubated at 37 °C in the dark for 30 minutes after which lipid peroxidation was initiated with either diethylmaleate (9 mM) for 2 hours or (1*S*,3*R*)-RSL3 (4 μM) for 6 hours. Treated TF1a cells were then collected by centrifugation at 300×*g* for 3–4 minutes, whereas treated HEK-293 cells were then washed by DPBS, detached with accutase followed by centrifugation at 300×*g* for 3–4 minutes. Cells were resuspended in DPBS and analyzed by flow cytometry at a final concentration of 1 × 10^6^ cells mL^–1^ (*λ*_ex_ = 488 nm; *λ*_em_ = 525 ± 25 nm). Cells that were not treated with DEM/RSL3 were used as negative control. Cells that were not treated with thiosulfinates were used as positive control.

### VI. Cell viability

TF1a cells were cultured in RPMI-1640 media with 10% FBS and 1% penicillin–streptomycin. HEK 293 cells were cultured in MEM media with 10% FBS, 1% non-essential amino acid, 1 mM sodium pyruvate and 1% penicillin–streptomycin. Cells (5 × 10^5^ cells mL^–1^) were treated with each of thiosulfinates **1**, **2** and **4** at final concentrations ranging from 5 μM to 200 μM and incubated at 37 °C for 22 hours in phenol red-free RPMI-1640 media with 10% FBS and 1% penicillin–streptomycin for TF1a cells and phenol red-free MEM media with 10% FBS, 1% non-essential amino acid, 1 mM sodium pyruvate and 1% penicillin–streptomycin for HEK 293 cells in a humidified atmosphere containing 5% CO_2_ in air. Treated HEK 293 cells were washed with DPBS followed by accutase to detach from the plate. Cells were collected into the flow cytometry tubes and then treated with 7-AAD (5 μL of a 50 μg mL^–1^ solution/1 × 10^6^ cells) for 10 minutes and analyzed by flow cytometry at a final concentration of 5 × 10^5^ cells mL^–1^ (10 000 events; *λ*_ex_ = 488 nm; *λ*_em_ = 675 ± 25 nm). Cell viability was also determined using hemocytometry with trypan blue. After incubation of TF1a cells (5 × 10^5^ cells mL^–1^) with each of **1**, **2** and **4** for 22 hours, 50 μL of 0.4% trypan blue solution was added into 350 μL of cells. The cells were counted using a hemocytometer under light microscopy. The cells which excluded the probe were considered viable. About 200–250 cells were counted for each sample.

### VII. Cellular thiol concentration

TF1a (5 × 10^5^ cells mL^–1^) and HEK-293 (5 × 10^5^ cells mL^–1^) cells were treated with each of the thiosulfinates **1**, **2** and **4** at final concentrations from 10 μM to 200 μM separately and incubated at 37 °C for 22 hours in phenol red-free media in a humidified atmosphere containing 5% CO_2_ in air. Cells were then lysed with HEPES hypotonic buffer and the cytosolic fraction was collected. Intracellular thiol concentration was determined by absorbance at 412 nm using the glutathione colorimetric assay by titration with Ellman's reagent (5,5′-dithio-bis-2-nitrobenzoic acid) using glutathione as a standard. Intracellular protein concentration was determined using a commercial Bradford assay kit.

### VIII. Papain inhibition assays^22^

Papain (25 μM) was incubated with DTT (1 mM) for 30 minutes at 0 °C in pH 6.1 buffer (2 mM EDTA, 50 mM sodium acetate). The small molecules were then removed by filtration through an 10 kDa Amicon Filter. Stock solutions of the various thiosulfinates in acetonitrile were prepared directly before use. The chromogenic substrate *N*α-benzoyl-dl-arginine 4-nitroanilide hydrochloride (BAPNA) was first dissolved in a minimal amount of DMSO and diluted in buffer to a final concentration of 1 mM. The wells of a 96 well microplate were loaded with buffer, papain solution (3.2 μM, 25 μL) and thiosulfinate solution to a final volume of 160 μL. The microplate was then incubated at 37 °C for 30 minutes. BAPNA (200 μM, 40 μL) was added by the reagent dispenser of the microplate reader and the activity of papain was measured at 37 °C by monitoring the production of *p*-nitroaniline by absorbance at 410 nm every 5 seconds for 5 minutes.

## Supplementary Material

Supplementary information
