# Supplementary material for: The medicinal thiosulfinates from garlic and Petiveria are not radical-trapping antioxidants in liposomes and cells, but lipophilic analogs are
Source: Chem Sci. 2015 Jul 29;6(11):6165–78. doi: 10.1039/c5sc02270c (PMC6054074; doi:10.1039/c5sc02270c)
Supplement: Supplementary file 1 [file SC-006-C5SC02270C-s001.pdf]

Electronic Supplementary Material (ESI) for Chemical Science.
This journal is © The Royal Society of Chemistry 2015

Supporting Information

for

The Medicinal Thiosulfinates from Garlic and Petiveria are Not Radical-Trapping Antioxidants, but Lipophilic Analogs Are

Bo Li, Feng Zheng, Jean-Philippe R. Chauvin and Derek A. Pratt*
Department of Chemistry and Biomolecular Sciences, University of Ottawa, Ottawa, Ontario K1N 6N5, Canada
dpratt@uottawa.ca

Table of Contents

Figure S1. Samples of MeOAMVN-mediated oxidations of liposomes and curve fitting for relative rates........S2

Table S1. Relative rate constants for the reactions of 9-triptycenesulfenic acid **3** and hexylated petivericin **4** with MeOAMVN-derived peroxyl radicals........S3

Figure S2. Hydroperoxide production in the oxidation of PLPC liposomes supplemented with hexylated petivericin **4** and NAC........S3

Figure S3. Decomposition of allicin **1** and petivericin **2**........S4

Figure S4. Decomposition of petivericin **2** in liposomes in the presence of NAC and formation of the corresponding mixed disulfide........S5

Figure S5. Decomposition of hexylated petivericin **4** in liposomes in the presence of NAC and formation of the corresponding mixed disulfide........S5

Figure S6. Representative dose-response curves on lipid peroxidation inhibition obtained from flow cytometry in HEK293 cells..S6

Figure S7. Representative dose-response curves obtained from papain inactivation assay.........S7

^{1}H and ^{13}C NMR spectra of compounds **5-10**..S8-13

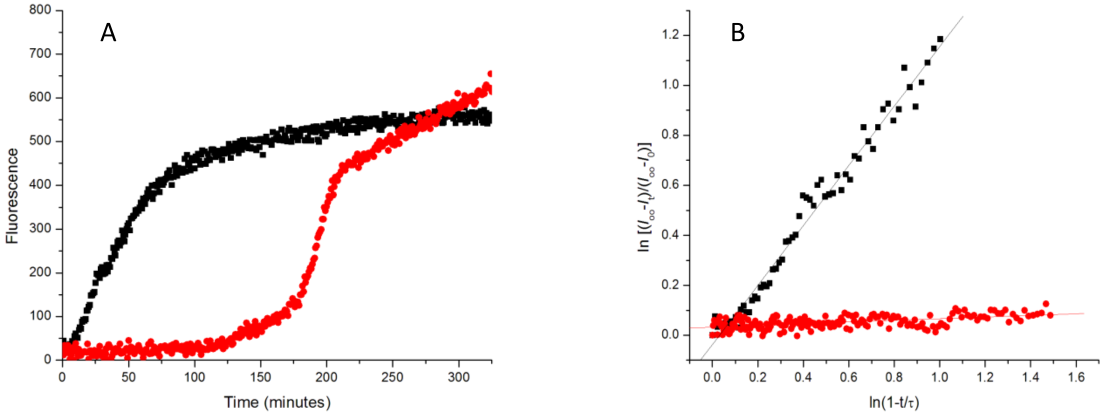

Figure S1. (A) Representative fluorescence (at 520 nm) intensity-time profiles from MeOAMVN-mediated (0.2 mM) oxidations of egg phosphatidylcholine liposomes (1 mM in PBS buffer, pH 7.4) containing 0.15 µM H_2B-PMHC and 4.5 µM of 9-triptycenesulfenic acid (red) and hexylated petivericin (black). (B) The data in (A) plotted according to Eq. (4) for 9-triptycenesulfenic acid (red) and hexylated petivericin (black).

Table S1. Relative rate constants for the reactions of 9-triptycenesulfenic acid (**3**) and hexylated petivericin (**4**) with MeOAMVN-derived peroxyl radicals derived from data in Figure 1C and 1D.

[Antioxidant]	$k_{inh}^{H2B\text{-}PMHC}/k_{inh}^{\mathbf{3}}$	$k_{inh}^{H2B\text{-}PMHC}/k_{inh}^{\mathbf{4}}$
4.5	0.040	0.948
9.0	0.043	1.134
13.5	0.042	1.192
18	0.039	1.071
22.5	0.036	0.965
**average**	**0.040±0.004**	**1.06±0.11**

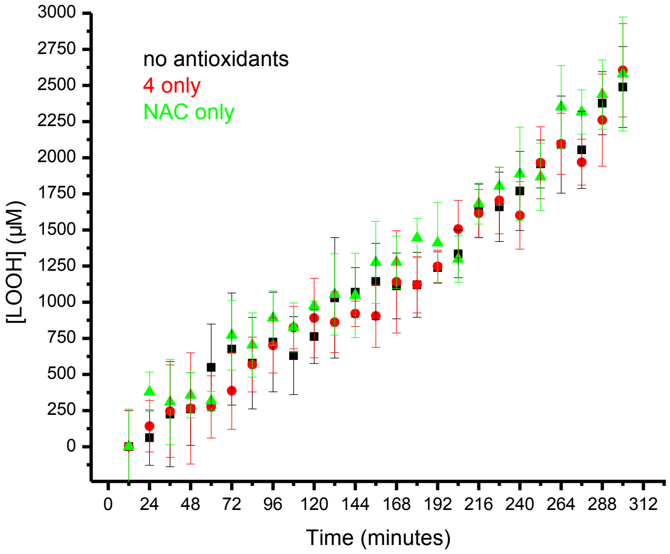

Figure S2. Hydroperoxide production in the oxidation of 1-palmitoyl-2-linoleyl-*sn*-glycero-3-phosphocholine liposomes (13.3 mM in phosphate-buffered saline, pH 7.4) initiated by MeOAMVN (150 μM) in the presence of 25 μM **4** only (red), 25 μM N-acetylcysteine only (green), or no additives (black).

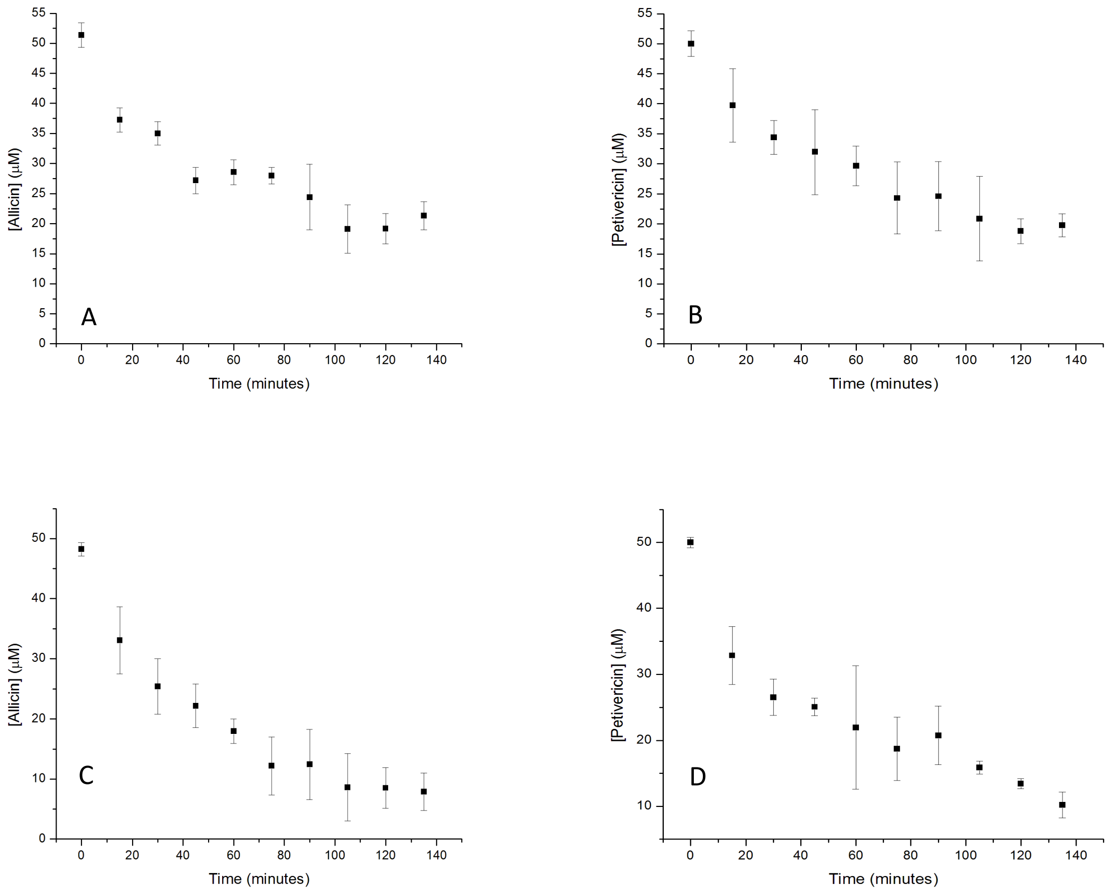

Figure S3. Decomposition of 50 μM allicin **1** without MeOAMVN (A) or with 0.2 mM MeOAMVN and 0.15 μM H_2B-PMHC (C); and Decomposition of 50 μM petivericin **2** without MeOAMVN (B) or with 0.2 mM MeOAMVN and 0.15 μM H_2B-PMHC (D) in unilamellar egg phosphatidylcholine liposomes.

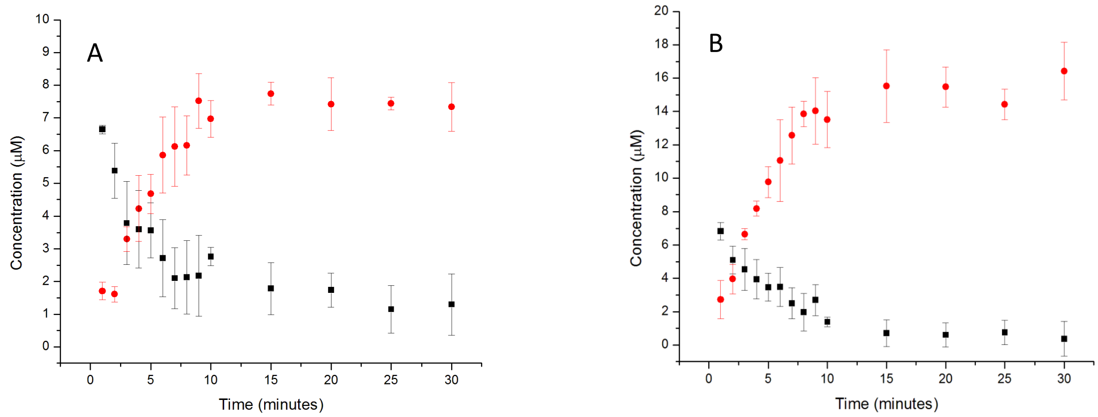

Figure S4. Decomposition of 7.5 µM petivericin **2** (■) in MeOAMVN-mediated (0.2 mM) oxidations of egg phosphatidylcholine liposomes (1 mM) containing 0.15 µM H_2B-PMHC and either 7.5 µM (A) or 15 µM NAC (B) in PBS buffer of pH 7.4 and formation of the corresponding mixed disulfide (●).

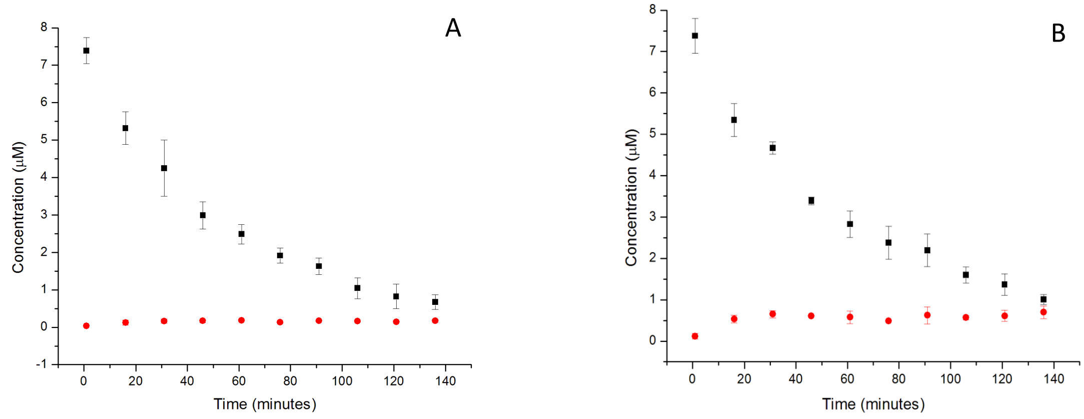

Figure S5. Decomposition of 7.5 µM hexylated petivericin **4** (■) in MeOAMVN-mediated (0.2 mM) oxidations of egg phosphatidylcholine liposomes (1 mM) containing 0.15 µM H_2B-PMHC and 7.5 µM (A) or 15 µM NAC (B) in PBS buffer of pH 7.4 and formation of the corresponding mixed disulfide (●).

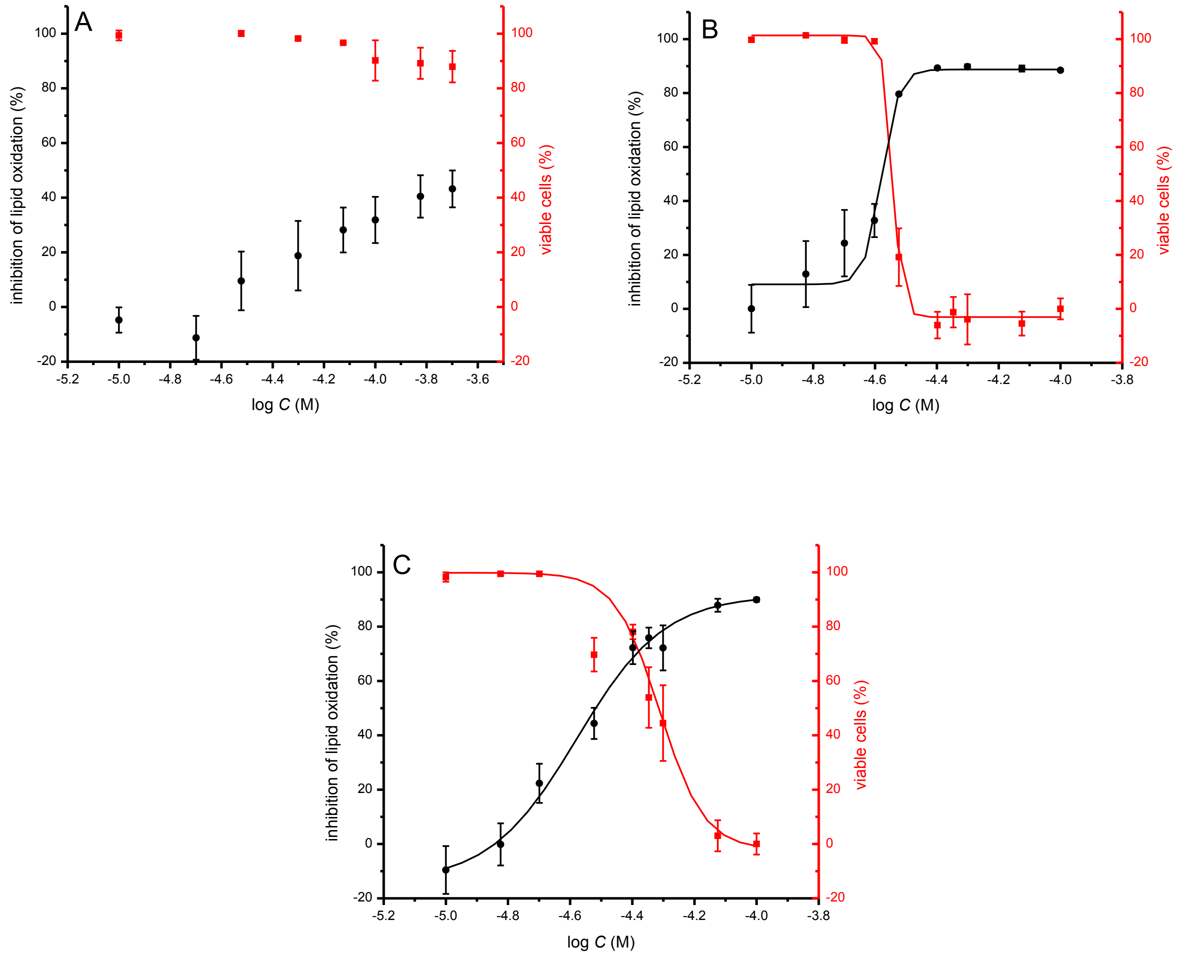

Figure S6. Representative dose-response curves obtained from flow cytometry (1×10^{6} cells/mL; λ_{ex} = 488 nm, λ_{em} = 525±25 nm; 10,000 events) following induction of oxidative stress with diethylmaleate (DEM, 9 mM) in HEK293 cells grown in MEM media containing either hexylated petivericin (**4**, A), allicin (**1**, B) or petivericin (**2**, C) (5-200 μM) for 22 hours at 37 °C. Cells were incubated with the lipid peroxidation reporter C11-BODIPY$^{581/591}$ (1 μM) for 30 minutes prior to DEM treatment.

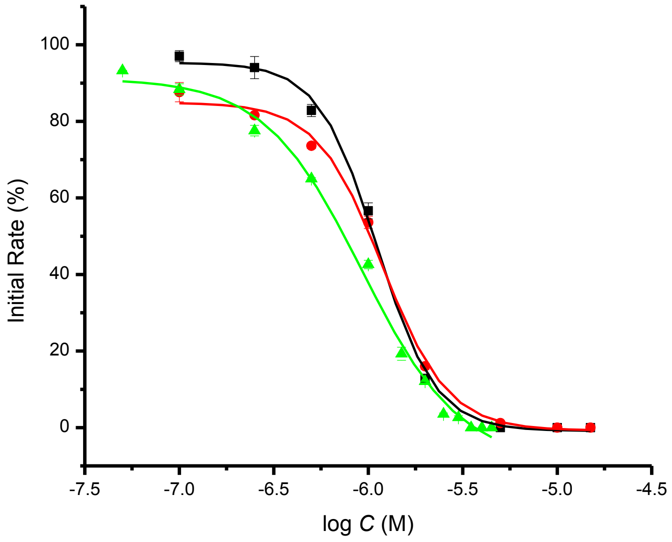

Figure S7. Representative dose-response curves obtained from papain inactivation assay in EDTA/sodium acetate buffer (pH 6.1) for hexylated petivericin (**4**, green; IC_{50} = 1.0±0.1 μM), allicin (**1**, red; IC_{50} = 1.2±0.2 μM) or petivericin (**2**, black; IC_{50} = 1.1±0.1 μM). Papain activity was determined by measuring the rate of increase of absorbance at 410 nm.

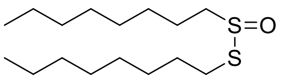
S=O
S

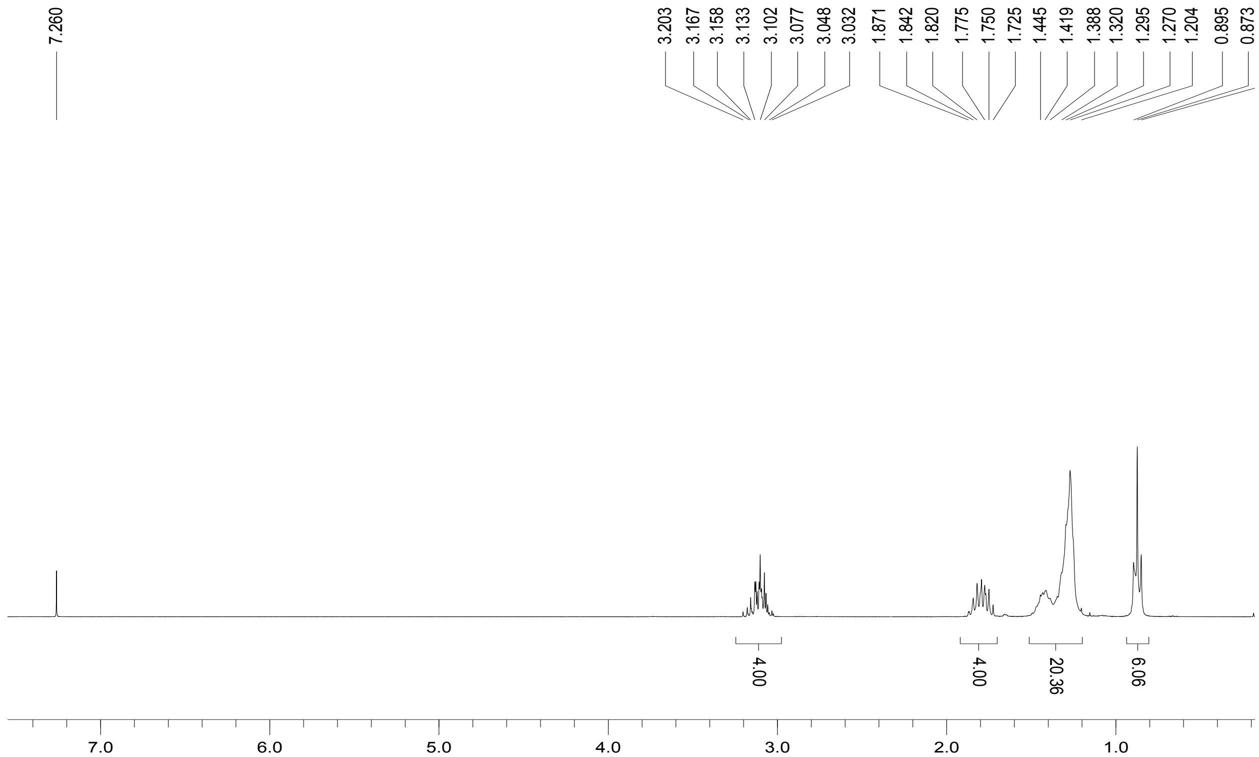
7.260
3.203
3.167
3.158
3.133
3.102
3.077
3.048
3.032
1.871
1.842
1.820
1.775
1.750
1.725
1.445
1.419
1.388
1.320
1.295
1.270
1.204
0.895
0.873
4.00
4.00
20.36
6.06
7.0
6.0
5.0
4.0
3.0
2.0
1.0
ppm (t1)

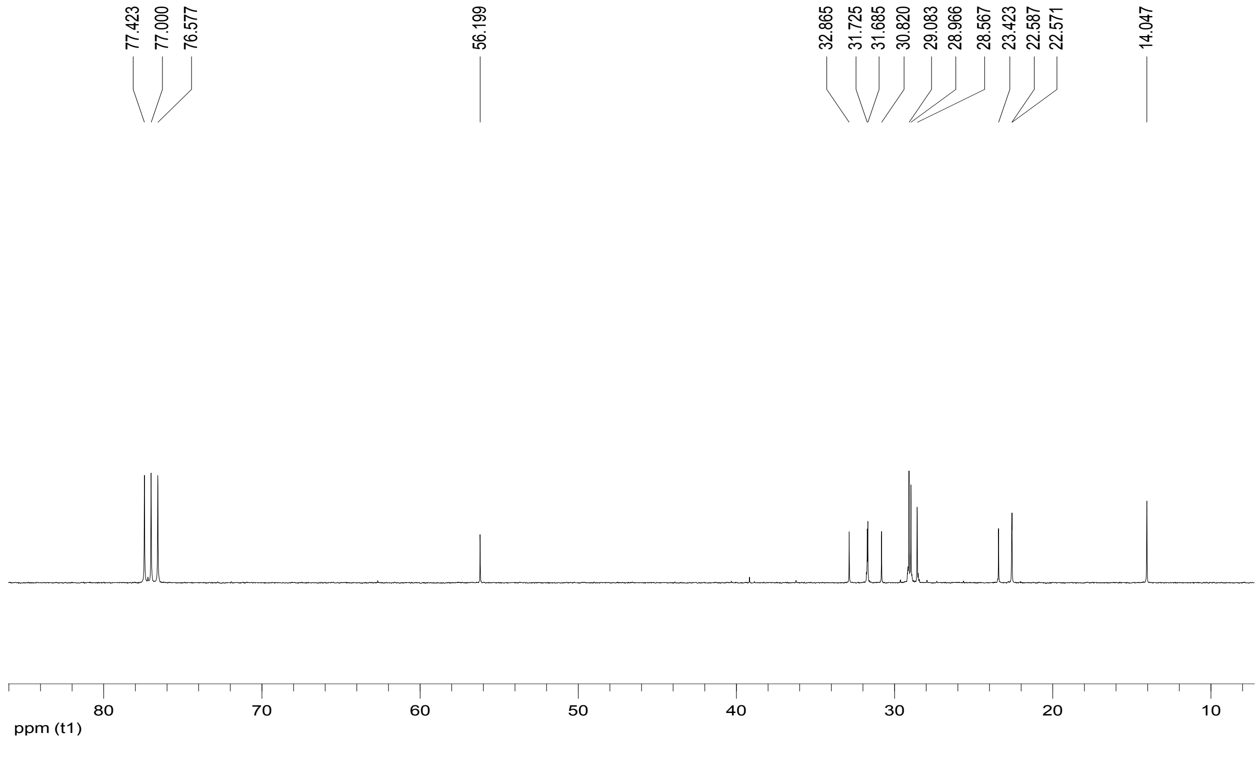
77.423
77.000
76.577
56.199
32.865
31.725
31.685
30.820
29.083
28.966
28.567
23.423
22.587
22.571
14.047
80
70
60
50
40
30
20
10
ppm (t1)

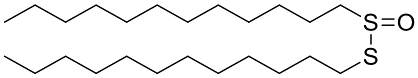
S=O
S

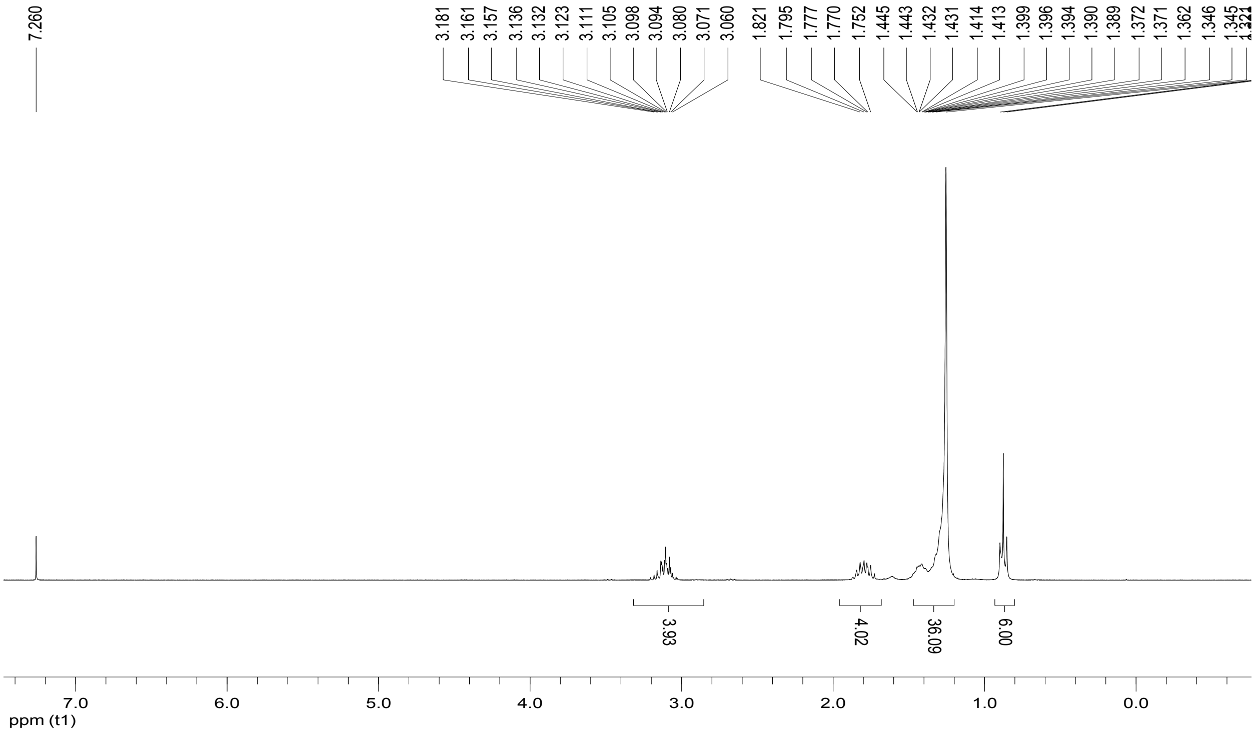
7.260
3.181 3.161 3.157 3.136 3.132 3.123 3.111 3.105 3.098 3.094 3.080 3.071 3.060
1.821 1.795 1.777 1.770 1.752 1.445 1.443 1.432 1.431 1.414 1.413 1.399 1.396 1.394 1.390 1.389 1.372 1.371 1.362 1.346 1.345
3.93
4.02
36.09
6.00
7.0 6.0 5.0 4.0 3.0 2.0 1.0 0.0
ppm (t1)

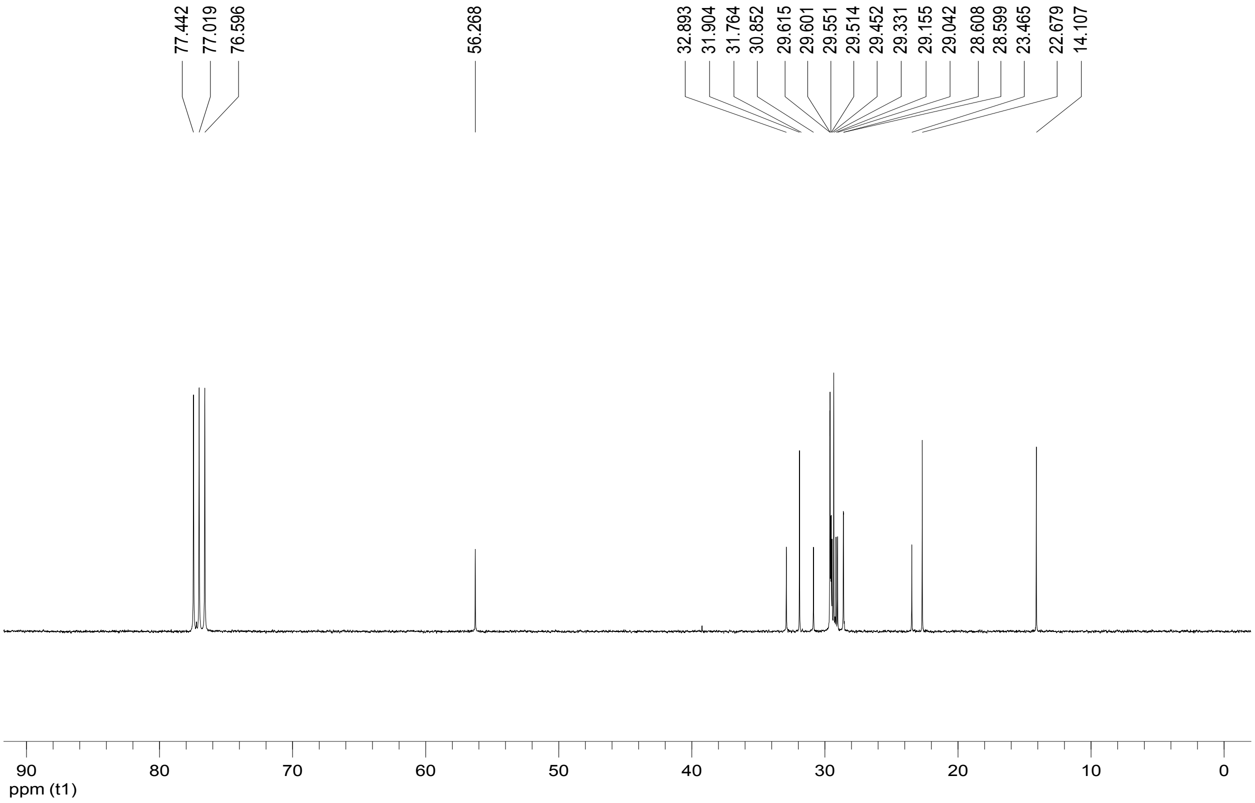
77.442 77.019 76.596
56.268
32.893 31.904 31.764 30.852 29.615 29.601 29.551 29.514 29.452 29.331 29.155 29.042 28.608 28.599 23.465 22.679 14.107
90 80 70 60 50 40 30 20 10 0
ppm (t1)

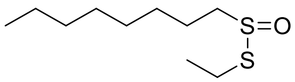
S=O
S

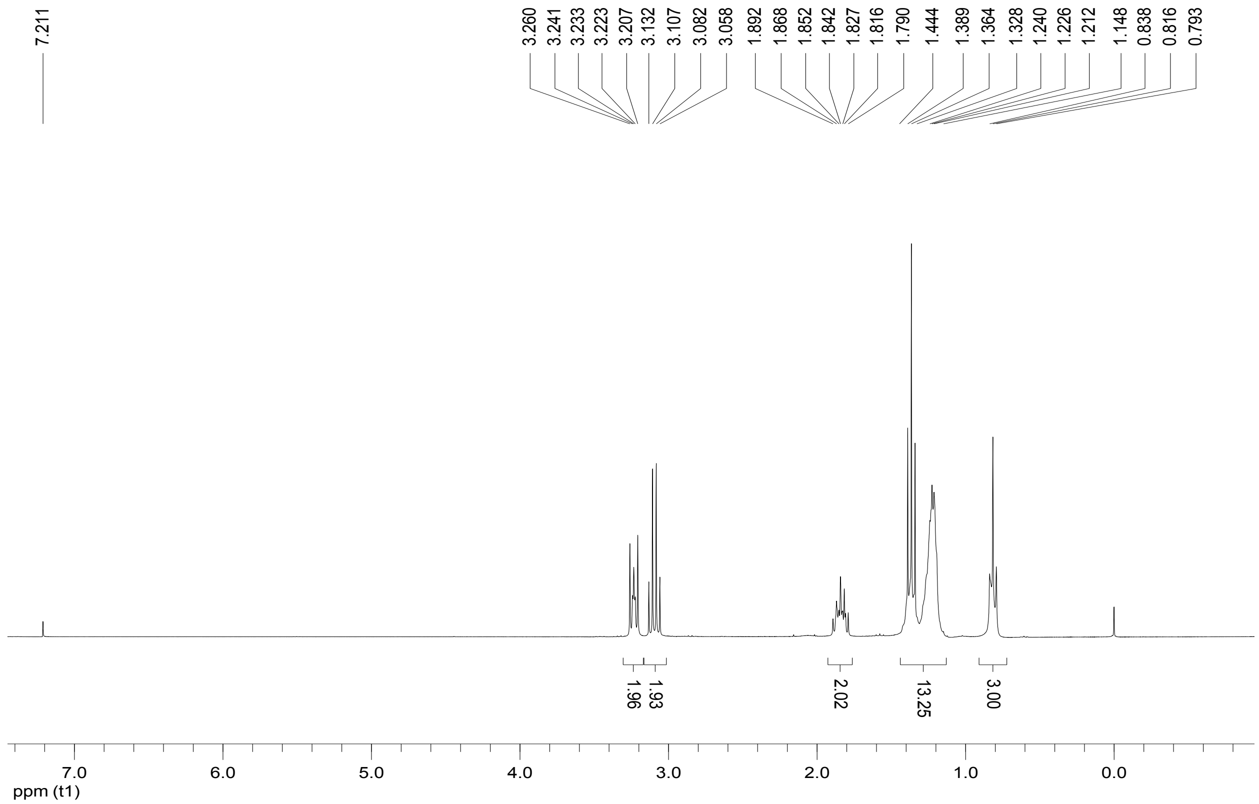
7.211
3.260
3.241
3.233
3.223
3.207
3.132
3.107
3.082
3.058
1.892
1.868
1.852
1.842
1.827
1.816
1.790
1.444
1.389
1.364
1.328
1.240
1.226
1.212
1.148
0.838
0.816
0.793
1.96
1.93
2.02
13.25
3.00
7.0
6.0
5.0
4.0
3.0
2.0
1.0
0.0
ppm (t1)

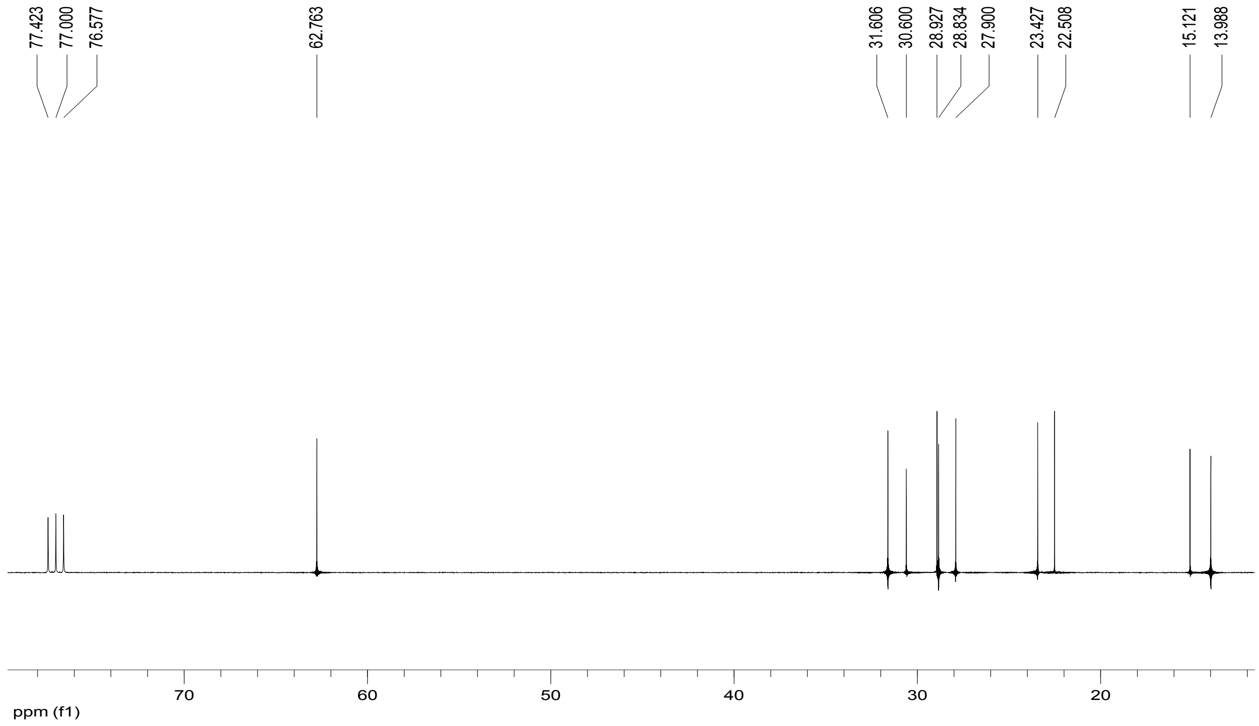
77.423
77.000
76.577
62.763
31.606
30.600
28.927
28.834
27.900
23.427
22.508
15.121
13.988
70
60
50
40
30
20
ppm (f1)

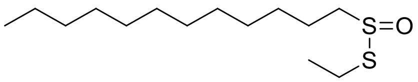
S=O
S

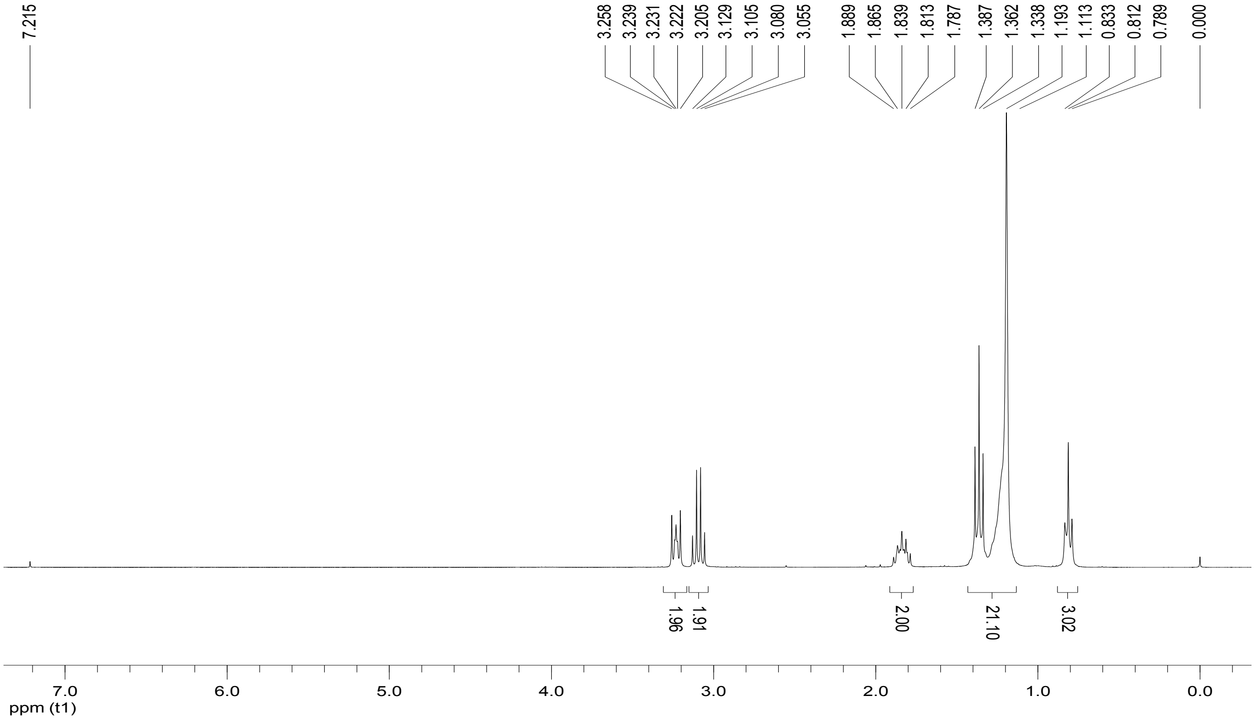
7.215
3.258
3.239
3.231
3.222
3.205
3.129
3.105
3.080
3.055
1.889
1.865
1.839
1.813
1.787
1.387
1.362
1.338
1.193
1.113
0.833
0.812
0.789
0.000
1.96
1.91
2.00
21.10
3.02
7.0
6.0
5.0
4.0
3.0
2.0
1.0
0.0
ppm (t1)

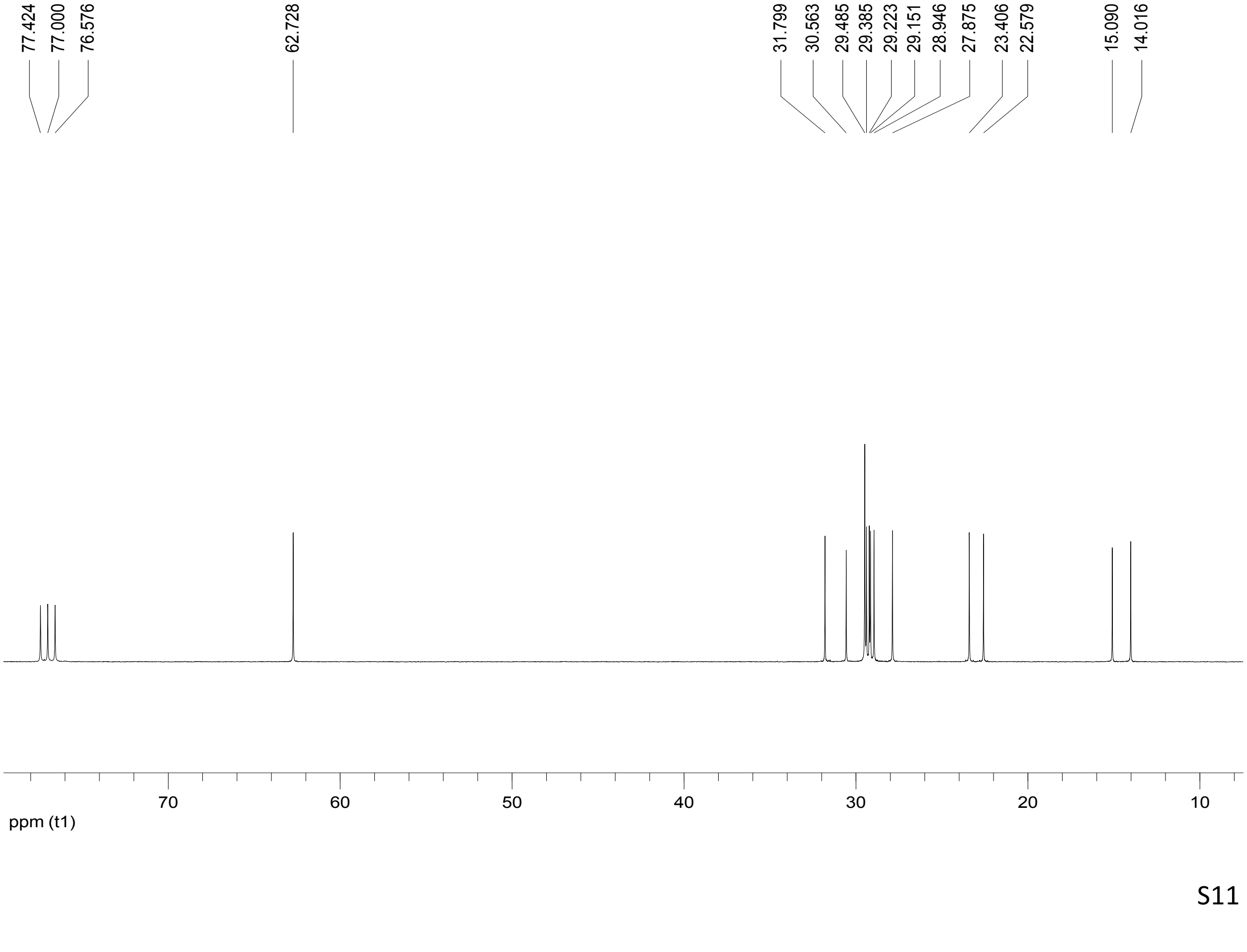
77.424
77.000
76.576
62.728
31.799
30.563
29.485
29.385
29.223
29.151
28.946
27.875
23.406
22.579
15.090
14.016
70
60
50
40
30
20
10
ppm (t1)

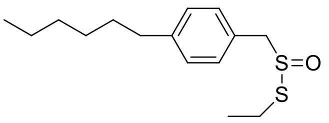
S=O
S

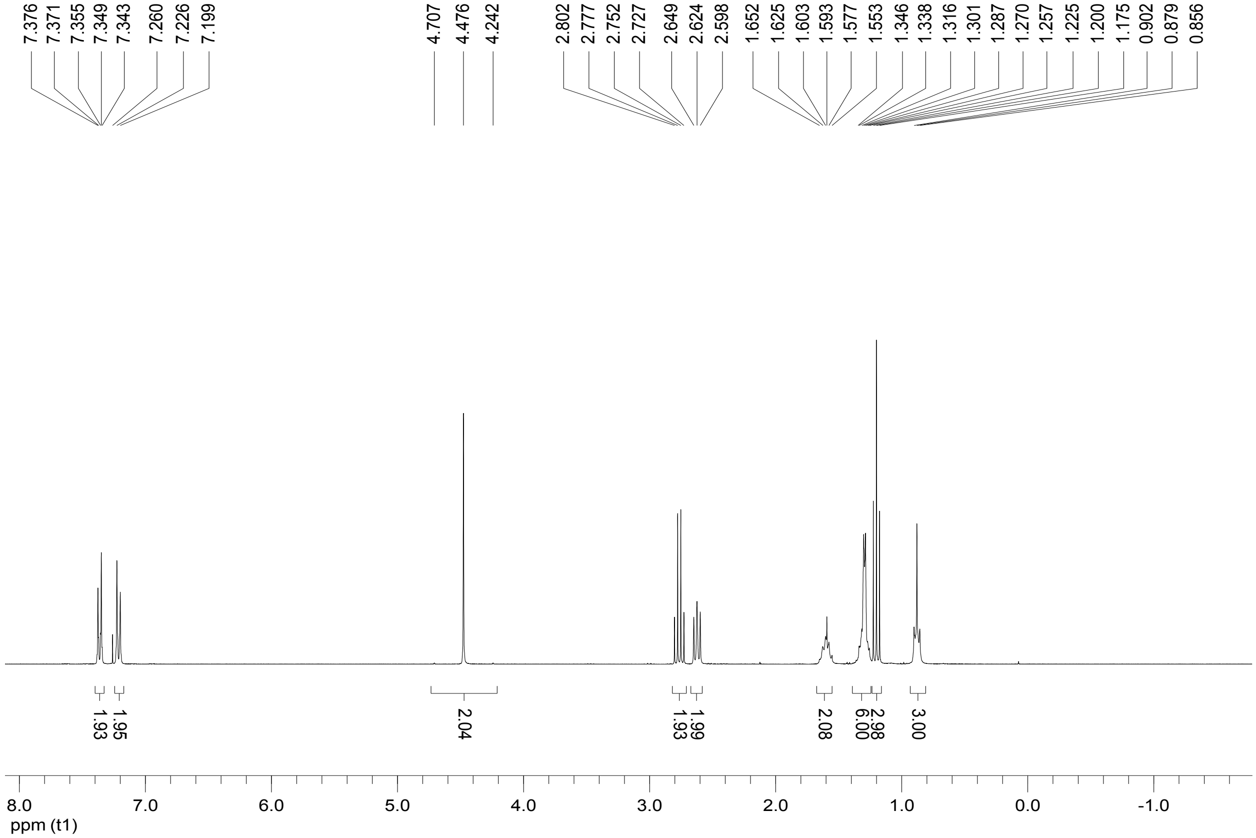
7.376
7.371
7.355
7.349
7.343
7.260
7.226
7.199
4.707
4.476
4.242
2.802
2.777
2.752
2.727
2.649
2.624
2.598
1.652
1.625
1.603
1.593
1.577
1.553
1.346
1.338
1.316
1.301
1.287
1.270
1.257
1.225
1.200
1.175
0.902
0.879
0.856
1.93
1.95
2.04
1.93
1.99
2.08
6.00
2.98
3.00
8.0
7.0
6.0
5.0
4.0
3.0
2.0
1.0
0.0
-1.0
ppm (t1)

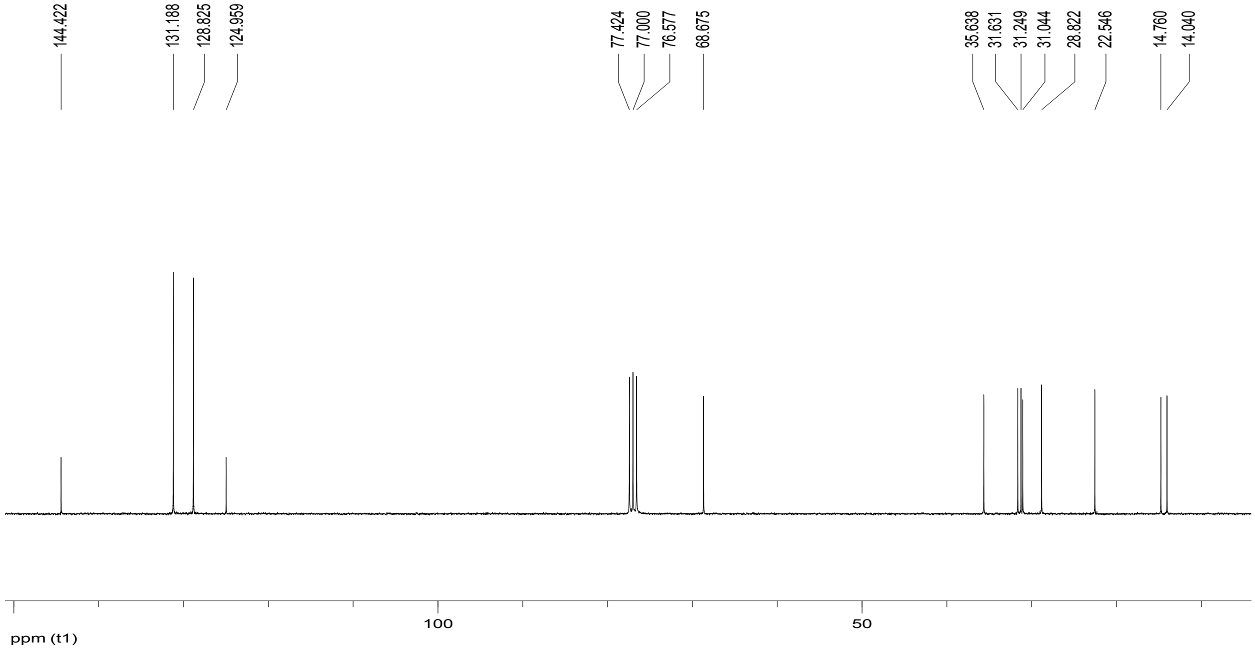
144.422
131.188
128.825
124.959
77.424
77.000
76.577
68.675
35.638
31.631
31.249
31.044
28.822
22.546
14.760
14.040
100
50
ppm (t1)

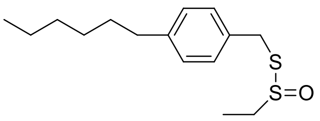
S
S=O

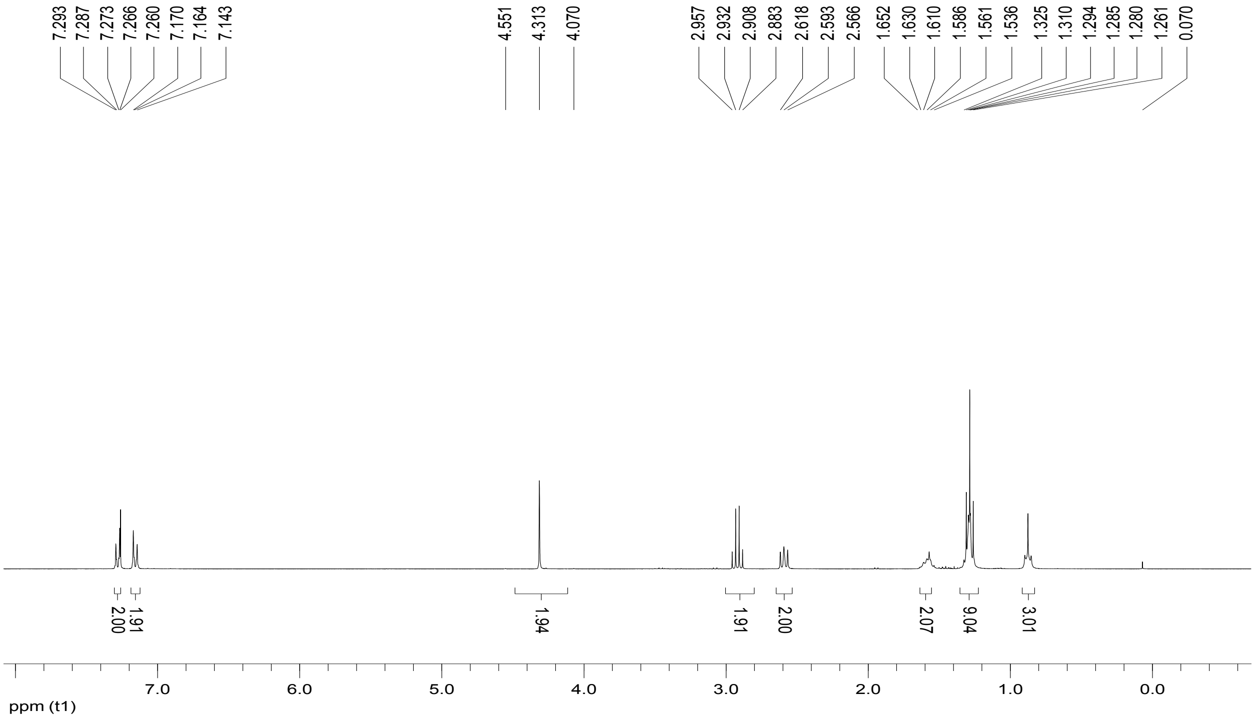
7.293
7.287
7.273
7.266
7.260
7.170
7.164
7.143
4.551
4.313
4.070
2.957
2.932
2.908
2.883
2.618
2.593
2.566
1.652
1.630
1.610
1.586
1.561
1.536
1.325
1.310
1.294
1.285
1.280
1.261
0.070
2.00
1.91
1.94
1.91
2.00
2.07
9.04
3.01
7.0
6.0
5.0
4.0
3.0
2.0
1.0
0.0
ppm (t1)

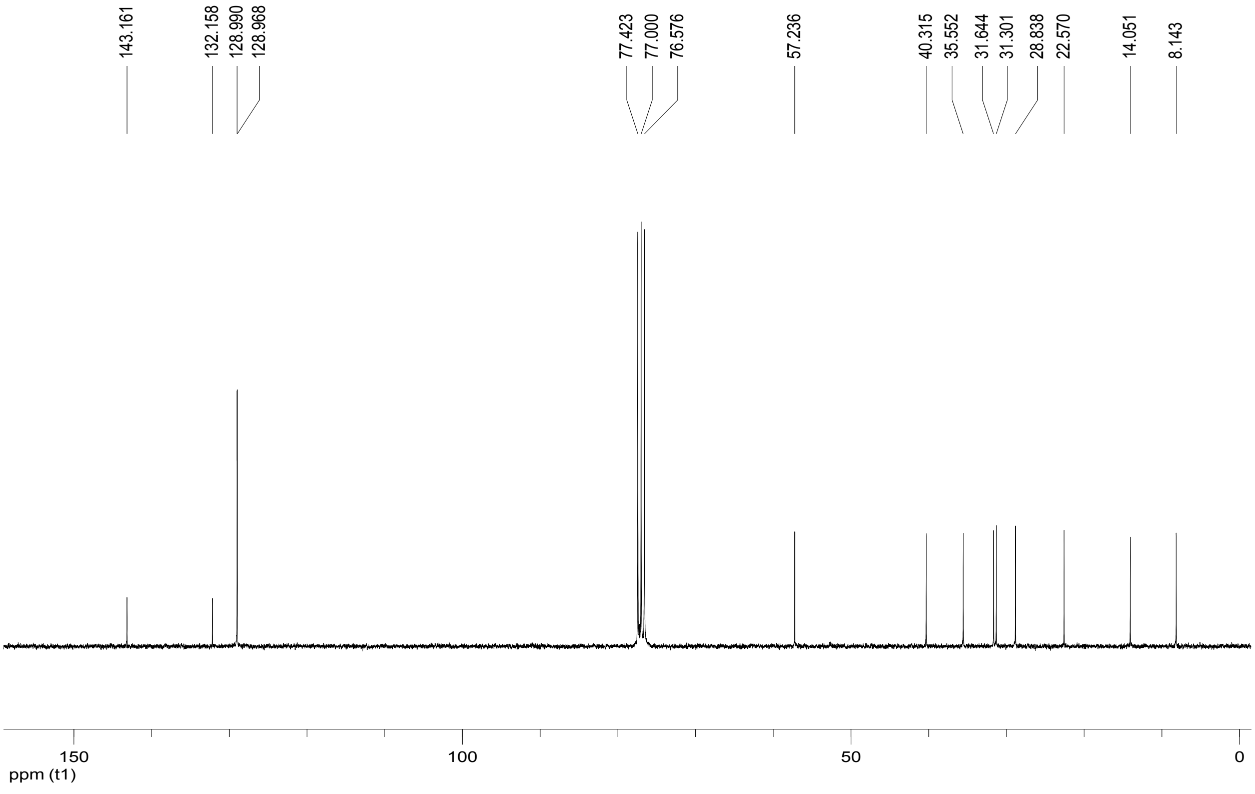
143.161
132.158
128.990
128.968
77.423
77.000
76.576
57.236
40.315
35.552
31.644
31.301
28.838
22.570
14.051
8.143
150
100
50
0
ppm (t1)
